# Impact of migration on the multi-strategy selection in finite group-structured populations

**DOI:** 10.1038/srep35114

**Published:** 2016-10-21

**Authors:** Yanling Zhang, Aizhi Liu, Changyin Sun

**Affiliations:** 1 School of Automation and Electrical Engineering, University of Science and Technology Beijing, Beijing 100083, China; 2 School of Automation, Southeast University, Nanjing 210096, China

## Abstract

For large quantities of spatial models, the multi-strategy selection under weak selection is the sum of two competition terms: the pairwise competition and the competition of multiple strategies with equal frequency. Two parameters *σ*_1_ and *σ*_2_ quantify the dependence of the multi-strategy selection on these two terms, respectively. Unlike previous studies, we here do not require large populations for calculating *σ*_1_ and *σ*_2_, and perform the first quantitative analysis of the effect of migration on them in group-structured populations of any finite sizes. The Moran and the Wright-Fisher process have the following common findings. Compared with well-mixed populations, migration causes *σ*_1_ to change with the mutation probability from a decreasing curve to an inverted U-shaped curve and maintains the increase of *σ*_2_. Migration (probability and range) leads to a significant change of *σ*_1_ but a negligible one of *σ*_2_. The way that migration changes *σ*_1_ is qualitatively similar to its influence on the single parameter characterizing the two-strategy selection. The Moran process is more effective in increasing *σ*_1_ for most migration probabilities and the Wright-Fisher process is always more effective in increasing *σ*_2_. Finally, our findings are used to study the evolution of cooperation under direct reciprocity.

Populations are usually divided into several subpopulations separated by geographical distance. Migration, linking subpopulations, is one of the oldest adaptation measures in the animal kingdom and human society. The effect of migration on developing such populations has attracted researchers from various fields. Masses of related analytic studies have been performed by geneticists and mathematicians to explain the correlation between genetic distance and geographic distance through the (one-dimensional or two-dimensional) stepping-stone models^1^,^2^,^3^,^4^,^5^. The update rules most extensively used are the frequency-independent Moran process and the frequency-independent Wright-Fisher process.

The above-mentioned studies assume that all individuals have the same fitness. Nonetheless, most realistic settings do not operate in this way, and individuals’ fitness is shaped by the behaviors of themselves together with those who live in the same environment. Evolutionary game theory provides a powerful mathematical framework to deal with such interactions^6^,^7^,^8^,^9^,^10^. Under the framework, spatial models have attracted more and more attention through games on graphs^11^,^12^,^13^,^14^,^15^,^16^,^17^ and populations comprised of subpopulations^18^,^19^,^20^,^21^,^22^,^23^,^24^,^25^,^26^,^27^. For a large class of spatial models, including games in phenotype space^28^, games on sets^29^, games on islands^30^, and games in group-structured populations^31^, the two-strategy selection and the multi-strategy selection under weak selection can be characterized by a single parameter^32^ and two parameters^33^, respectively. Weak selection, meaning the fitness varies little among individuals, has been widely used for analytical studies^34^,^35^,^36^,^37^,^38^,^39^,^40^,^41^,^42^. Prior research has showed that the results under weak selection, in general, could not be extrapolated to strong selection^43^,^44^. There are two reasons why weak selection is still so popular is as follows: Weak selection makes it possible to obtain analytical results without additional assumptions; Weak selection is a natural situation in which a particular game makes a small contribution on the overall fitness of individuals, for example, (a) strategies are similar (as in the adaptive dynamics^45^,^46^), (b) individuals get confused about payoffs during the strategy update.

When a game between two strategies (say 1, 2) described by the payoff matrix (*a*_*ij*_)_2 × 2_ (*a*_*ij*_ is the payoff of an individual using *i* when interacting with an individual using *j*) is considered^32^, strategy 1 is more abundant than strategy 2 on average under weak selection if
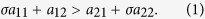


The parameter *σ* quantifies the influence of the population structure (including the update rule) on the two-strategy selection. When a game among *S* ≥ 3 strategies (say 1, 2, …, *S*) described by the payoff matrix (*a*_*ij*_)_*S*×*S*_ (similar to (*a*_*ij*_)_2×2_) is considered^33^, the average frequency of strategy *k* ∈ {1, 2, …, *S*} over the stationary distribution is greater than 1/*S* under weak selection if


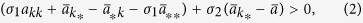


where 
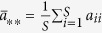
, 
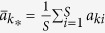
, 
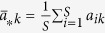
, and 
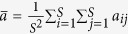
. It indicates that the multi-strategy selection is simply the sum of two competition terms. The first term, 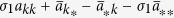, demonstrates an average over all pairwise comparisons between strategy *k* and any other strategies, and the second, 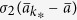, the competition of all *S* strategies when they have the equal frequency 1/*S*. Accordingly, the parameters *σ*_1_ and *σ*_2_ quantify the effect of the population structure on the pairwise competition and the competition of all strategies with equal frequency, respectively. Moreover, they quantify the dependence of the multi-strategy selection on these two competition terms, respectively.

It has been showed that the above-mentioned parameters *σ*, *σ*_1_, and *σ*_2_ do not depend on the entries of the payoff matrix but rely on the population structure (including the update rule)^32^,^33^. Two previous studies have investigated the effect of migration on *σ* in large finite populations^30^ and any finite populations^47^, respectively. The values of *σ*_1_ and *σ*_2_ have been obtained for large finite populations^33^, but it is still unknown how varying migration patterns affect them in populations of any finite sizes.

In this paper, we will calculate the concrete values of *σ*_1_ and *σ*_2_ for group-structured populations of any finite sizes. Our study will proceed for the frequency-dependent Moran process (hereafter called the Moran process without ambiguity) and the frequency-dependent Wright-Fisher process (hereafter called the Wright-Fisher process). The Moran process represents an idealized case of overlapping generations and the Wright-Fisher process is a perfect case of non-overlapping generations. The realistic society cannot be fully depicted by either of them, and yet maybe by something in between. Under the assumption of large populations, it is known that the calculation procedures of *σ*_1_ and *σ*_2_ are the same for the Moran and the Wright-Fisher process when the same symbol represents the product of the population size and the mutation probability in the former and twice the product in the latter^28^. However, the two calculation procedures vary significantly in populations of any finite sizes and will be separately given in Supplementary Information. The key point for obtaining *σ*_1_ and *σ*_2_ is to calculate some special probabilities under neutral selection^33^. The corresponding probabilities for the Moran process have been derived in group-structured populations of any finite sizes^31^, and they will be applied to our model to get *σ*_1_ and *σ*_2_. Meanwhile for the Wright-Fisher process, we will acquire the corresponding probabilities for group-structured populations of any finite sizes, and then use them to calculate *σ*_1_ and *σ*_2_. For either of the two processes, the expressions of *σ*_1_ and *σ*_2_ given later hold for any ‘isotropic’ migration patterns, and a particular migration pattern fully captured by the migration range will be employed to clarify how the migration range impacts *σ*_1_ and *σ*_2_. We will compare the qualitative and the quantitative effect of the two processes on *σ*_1_ and *σ*_2_. Finally, our findings will be used to study the evolution of cooperation under direct reciprocity by considering the competition of *ALLC*, *ALLD*, *TFT*.

## Results

### Model description

Consider a group-structured population of size *N* which is fragmented into *M* groups (subpopulations). A group can be understood as an island in population genetics, and a particular company or a living community in human society. An individual adopts one of *S* strategies labelled as 1, 2, …, *S* and the payoff matrix is given by (*a*_*ij*_)_*S*×*S*_, where *a*_*ij*_ is the payoff of an individual using *i* against an individual using *j*. An individual only plays the game with all others of the same group. Interactions produce the payoff of an individual (say *k*), *p*_*k*_, and further his fitness, *f*_*k*_ = 1 + *δp*_*k*_, where *δ* ≥ 0 is the selection intensity. In this paper, the case of weak selection, *δ* → 0, is our focus.

The Moran process and the Wright-Fisher process will be analyzed, respectively. In the Moran process, all individuals of the population compete to reproduce one offspring proportional to their fitness, and then one individual is equi-probably chosen from the whole population to die. In the Wright-Fisher process, all individuals compete to reproduce *N* (population size) offspring proportional to their fitness, and the whole population is replaced by all the newborn offspring. Mutation or migration may happen to the offspring. An offspring mutates with probability *u* and then he follows the pattern of ‘global mutation’ to choose one strategy, i.e., one of *S* strategies is chosen equi-probably; Otherwise (with probability 1 − *u*), the offspring inherits the strategy of his parent. An offspring migrates with probability *v* and then he moves to one group according to a pre-defined migration pattern; Otherwise (with probability 1 − *v*), the offspring remains in his parent’s group.

**Figure 1:**
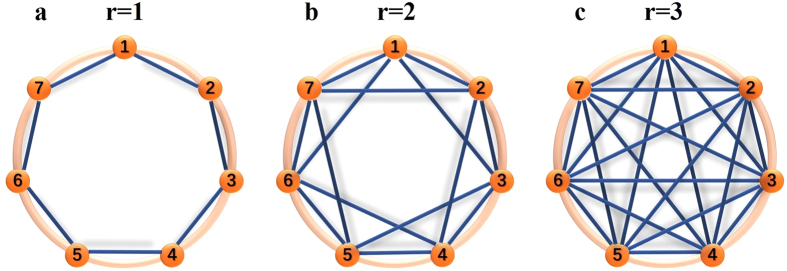
Migration patterns characterized by the migration range *r*. Seven groups (orange nodes) are arranged in a regular circle and labelled from 1 to 7 in clockwise. An edge exists between two nodes if and only if there is a potential single-step migration path between them. In other words, an offspring can migrate to one of the nodes connected to the node in which his parent is located. The distance between two groups takes on one of the values 1, 2, 3. The migration range *r* means that the displacements generated by a single-step migration form the set Ω(*r*) = {1, …, *r*} whose elements are performed equiprobably.

An undirected graph is used to illustrate the pattern of migration among the *M* groups arranged in a circle. Each node represents a group and an edge is a potential single-step migration path. We focus on the case of vertex-transitive graphs (with or without self-circle), which are homogeneous in the sense that they look the same from every node. Such migration patterns are ‘isotropic’ and have been widely investigated^1^,^2^,^3^,^4^,^5^,^30^,^31^. Examples include ‘local migration’ in which a single-step migration occurs equi-probably among all pairs from neighboring nodes or ‘global migration’ in which a single-step migration is equally likely to take individuals to any other nodes^30^. In this paper, the concrete values of *σ*_1_ and *σ*_2_ will be obtained for any ‘isotropic’ migration patterns, and then a particular migration pattern fully captured by the migration range *r* (Fig. 1) will be analyzed. The migration range *r*, which takes on one of the values 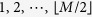 (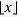 is the greatest integer not greater than *x*), means that all possible displacements generated by a single-step migration form the set Ω(*r*) = {1, 2, …, *r*} whose elements are performed equi-probably. The above-mentioned ‘local migration’ and ‘global migration’ can be characterized by *r* = 1 and 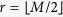, respectively.

In the above model, migration occurs after reproduction. We also consider a second case in which migration occurs before reproduction and the rest follows from the procedure above. Specifically, one individual is chosen equi-probably (from the whole population) to migrate before reproduction in the Moran process, and all individuals migrate before reproduction in the Wright-Fisher process. The corresponding results are qualitatively similar to but quantitatively different from those of the initial case by Monte-Carlo simulations (Supplementary Information), and will not be given in the main text. The quantitative difference emerges because mutation and migration in the second case cannot happen to the same individual during a generation, which happens in the initial case.

### The Moran process

The parameters *σ*_1_ and *σ*_2_ can be expressed by the probabilities (under neutral selection) assigned to the event that three randomly chosen (without replacement) individuals use given strategies and locations (see Methods for the detailed calculations). The expression of such probabilities has been derived for any mutation patterns and migration patterns^31^. Applying the expression to ‘global mutation’ of our model (see Supplementary Information: III and Methods for the detailed calculations), we have *σ*_1_ and *σ*_2_ of the Moran process denoted by 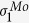 and 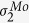 in the following expressions in which Φ_*i*_(*f*(*x*)) and Ψ_*i*_(*f*(*x*)) are abbreviated as Φ_*i*_ and Ψ_*i*_.


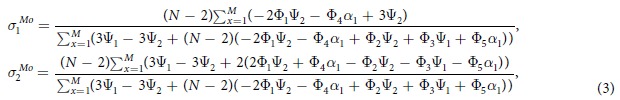


where 
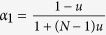
, 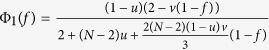, 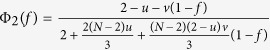, 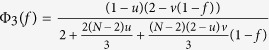, 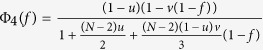, 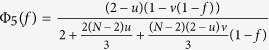, 
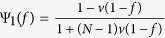
, 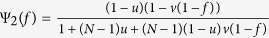.

The expressions hold for any ‘isotropic’ migration patterns described by *f*(*x*). To better clarify how the migration range affects *σ*_1_ and *σ*_2_, we focus on a representative type of migration patterns characterized by the migration range *r* (Fig. 1) whose corresponding *f*(*x*) is
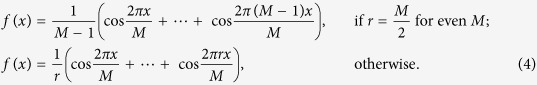


**Figure 2:**
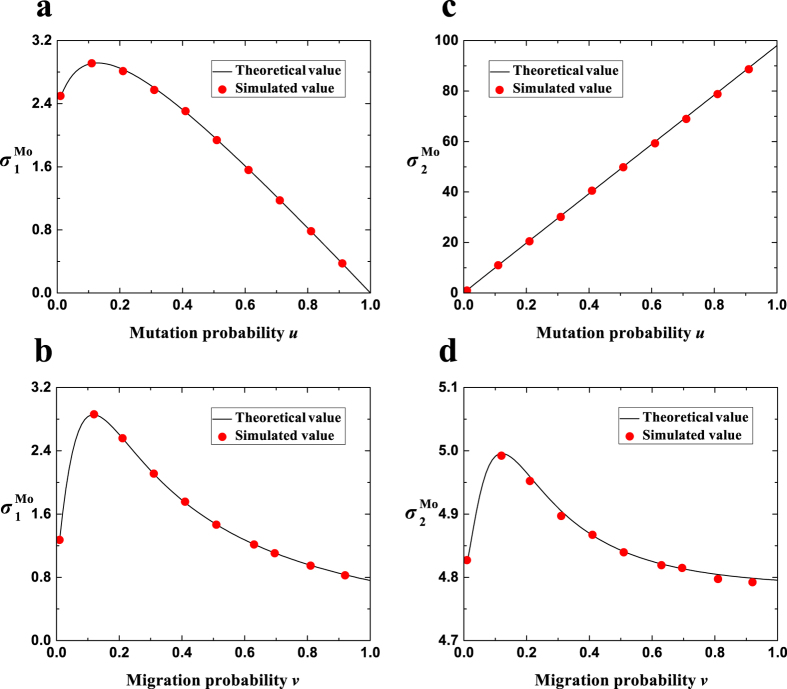
The theoretical values of 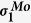 and 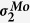 are in agreement with the simulated values. The solid line describes the theoretical values of 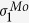 (**a**,**b**) or of 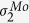 (**c**,**d**). The square denotes the simulated values of 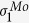 (**a**,**b**) or of 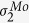 (**c**,**d**) averaged over 10^9^−10^6^ generations (starting to record at generation 10^6^). Parameters: (**a**,**c**) *v* = 1, *N* = 100, *M* = 19, *r* = 1; (**b**,**d**) *u* = 0.1, *N* = 50, *M* = 9, *r* = 4.

Besides the migration pattern, the expressions have no limitations on the non-zero mutation probability, the migration probability, the population size, or the group number. Figure 2 shows that the theoretical values of 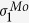 and 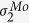 agree well with the simulated values for different mutation probabilities (*u*), migration probabilities (*v*), population sizes (*N*), group numbers (*M*), and migration ranges (*r*). It is noteworthy that the number of strategies *S* does not appear in the expressions of 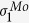 and 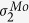, which agrees with the known conclusion that they are independent of *S*^33^.

After simple calculations, we have 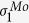 and 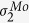 in the limit *u* → 0 as
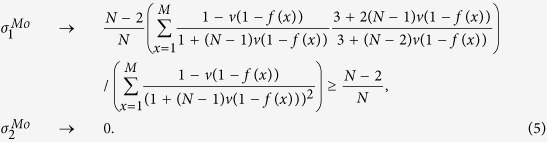


The pairwise competition plays an overriding role in determining the multi-strategy selection for extremely low mutation probabilities. It is intuitive since there exist simultaneously at most two strategies in the population. By letting *u* = 1 in equation (3), we get 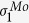 and 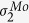 for *u* = 1 as
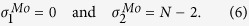


The competition of multiple strategies with equal frequency plays an overriding role in affecting the multi-strategy selection for sufficiently large populations and mutation probabilities (the condition for strategy *k* to be favored becomes 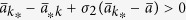).

It is easy to calculate 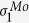 and 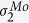 for *v* = 0 as
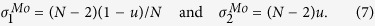


As the mutation probability *u* increases, the pairwise competition fades out of the multi-strategy selection and the competition of multiple strategies with equal frequency gradually dominates the multi-strategy selection. In the absence of migration (*v* = 0), the long-term population, in which the absorbing state is that all individuals are located in one group, evolves just like the well-mixed population. Therefore, equation (7) also gives the values of 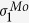 and 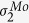 for the well-mixed population. For large populations, 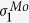 and 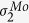 are approximated as 1 and *Nu* respectively, which is in agreement with the previous study^48^.

**Figure 3:**
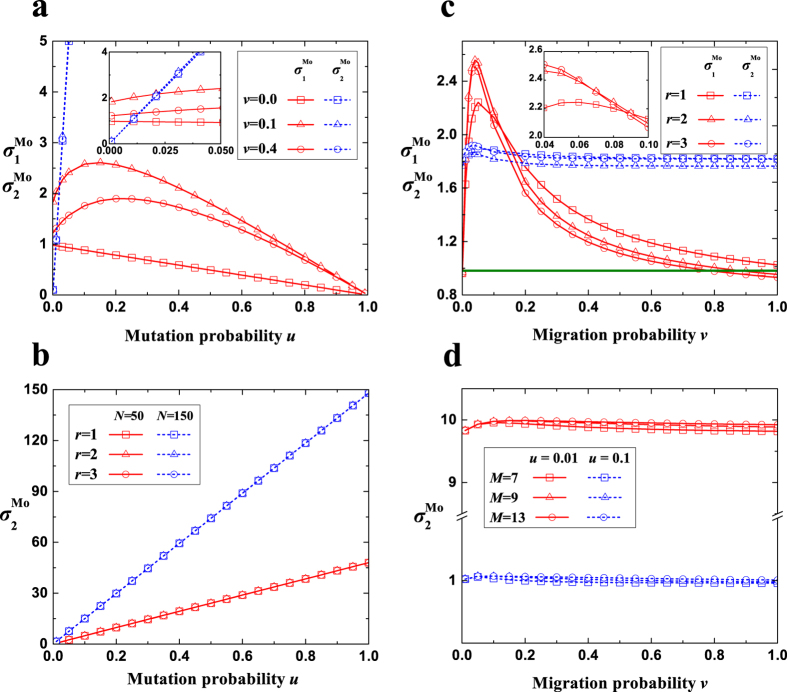
The changing trends of 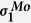 and 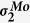. (**a**) As *u* increases, 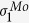 decreases for low *v* and high *v* (not shown), and exhibits an inverted U-shaped curve for moderate *v*. 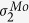 expands quickly as *u* increases. For very low *u*, 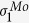 is greater than 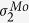 (inset). For a little higher *u*, 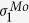 is smaller than 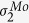, and the difference will expand quickly as *u* increases. (**b**) As *u* increases, 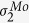 expands nearly linearly with a high speed of around *N*. *r* leads to a negligible change of 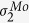. (**c**) *v* or *r* results in a significant change of 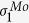 but a relatively negligible change of 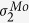. A moderate *v* maximizes 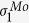, and most values of *v* near 0 produce much larger 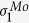 than that of the well-mixed population (solid line). The value of *r* corresponding to the maximum value of 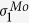 varies with *v* (inset). (**d**) 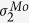 is maintained around a constant value (*Nu*) with the increase of *v* irrespective of *M*. Parameters: (**a**) *v* = 0.1, *M* = 7, *N* = 100; (**b**) *v* = 0.1, *M* = 7; (**c**) *u* = 0.018, *M* = 7, *N* = 100; (**d**) *N* = 100, *r* = 1.

When there exists migration (*v* ≠ 0), the comparison of 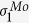 and 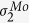 (the dependence of the multi-strategy selection on the pairwise competition and the competition of multiple strategies with equal frequency) is still mainly determined by the mutation probability *u* (Fig. 3a,b). In contrast to the well-mixed population (or *v* = 0), in which 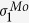 decreases monotonically with the increase of *u*, migration (moderate migration probabilities) causes the change of 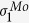 with respect to *u* to exhibit an inverted U-shaped curve. Similar to the well-mixed population (or *v* = 0), the value of 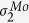 is still proportional to *u* with a coefficient around the population size *N*. This verifies the previous conjecture^33^ that *σ*_1_ ≪ *σ*_2_ holds for large *Nu*. Low mutation probabilities which are extremely close to zero lead to 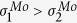, which means that the pairwise competition has an advantage over the competition of multiple strategies with equal frequency in determining the multi-strategy selection. Whereas the remaining vast majority of mutation probabilities result in 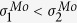, which means that the competition of multiple strategies with equal frequency gains the advantage over the pairwise competition.

We now focus on the effect of migration (probability and range) on 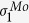 and 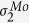 (Fig. 3). The migration probability or the migration range leads to a significant change of 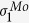 and a relatively negligible one of 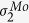. There exists a moderate migration probability maximizing 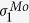, and the majority of migration probabilities near 0 result in much greater values of 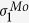 than that of the well-mixed population. The migration range which gives rise to the maximum value of 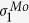 varies with the migration probability: it is the longest range 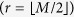 for very low migration probabilities, intermediate ranges for a little higher but still a small proportion of migration probabilities, and the shortest range (*r* = 1) for the remaining majority of migration probabilities.

### Comparing the Wright-Fisher process with the Moran process

Similar to the Moran process, the parameters *σ*_1_ and *σ*_2_ of the Wright-Fisher process can be expressed by the probabilities (under neutral selection) assigned to the event that three randomly chosen (without replacement) individuals use given strategies and locations. We obtain such probabilities of the Wright-Fisher process (see Supplementary Information: IV for the detailed calculations) for any mutation patterns and migration patterns following the example of the Moran process^31^. Applying these probabilities to ‘global mutation’ of our model (see Supplementary Information: V and Methods for the detailed calculations), we have *σ*_1_ and *σ*_2_ of the Wright-Fisher process which are denoted by 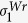 and 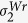 in the following expressions where 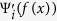 and 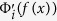 are abbreviated as 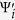 and 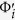.
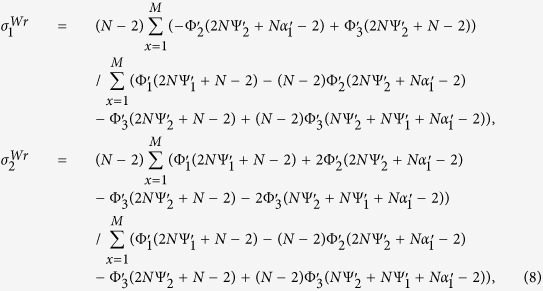


where 
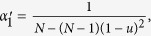
 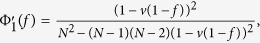

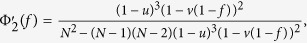

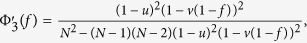
 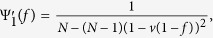

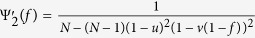
.

**Figure 4:**
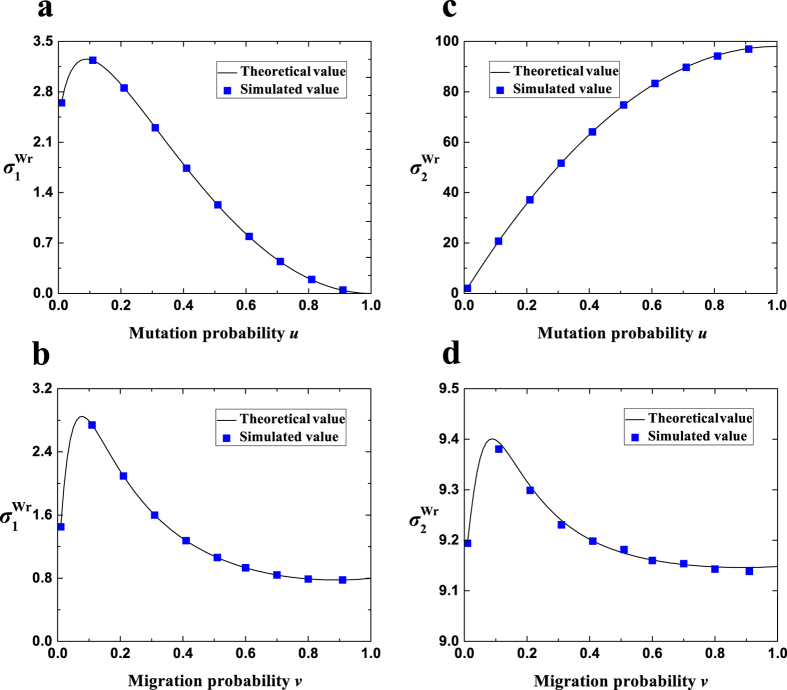
The theoretical values of 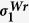 and 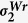 are in agreement with the simulated values. The solid line describes the theoretical values of 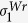 (**a**,**b**) or of 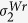 (**c**,**d**), and the square denotes the simulated values of 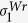 (**a**,**b**) or of 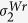 (**c**,**d**) averaged over 10^9^ − 10^6^ generations (starting to record at generation 10^6^). Parameters: (**a**,**c**) *v* = 0.1, *N* = 100, *M* = 19, *r* = 1; (**b**,**d**) *u* = 0.1, *N* = 50, *M* = 9, *r* = 4.

The expressions are suitable for any ‘isotropic’ migration patterns (*f*(*x*)), non-zero mutation probabilities (*u*), migration probabilities (*v*), population sizes (*N*), and group numbers (*M*), and have been verified by Monte Carlo simulations in Fig. 4. Additionally, they do not involve the number of strategies (*S*), which is in line with the previous literature^33^.

After simple calculations, we have
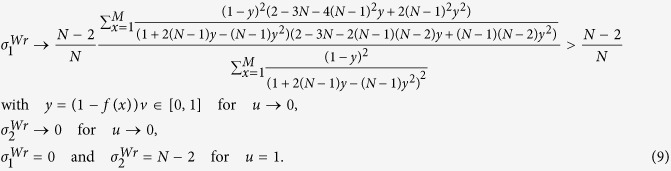
Just like the Moran process, the pairwise competition dominates exclusively the multi-strategy selection for extremely low mutation probabilities, and the competition of multiple strategies with equal frequency for sufficiently large populations and mutation probabilities.

For the group-structured population without migration (*v* = 0) or the well-mixed population,
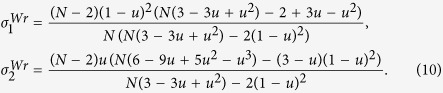


In the limit *N* → +∞, we have 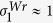 (0_*th*_ Taylor expansion) which is identical to the Moran process and 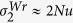 (1_*st*_ Taylor expansion) indicating that 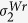 is twice 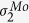 (of the Moran process). The result is in accordance with the previous literature^48^, since the expressions of *σ*_1_ and *σ*_2_ are the same for the Moran and the Wright-Fisher process in the sense that *Nu* in the former and 2*Nu* in the latter are denoted by the same symbol when the population is sufficiently large^28^.

**Figure 5:**
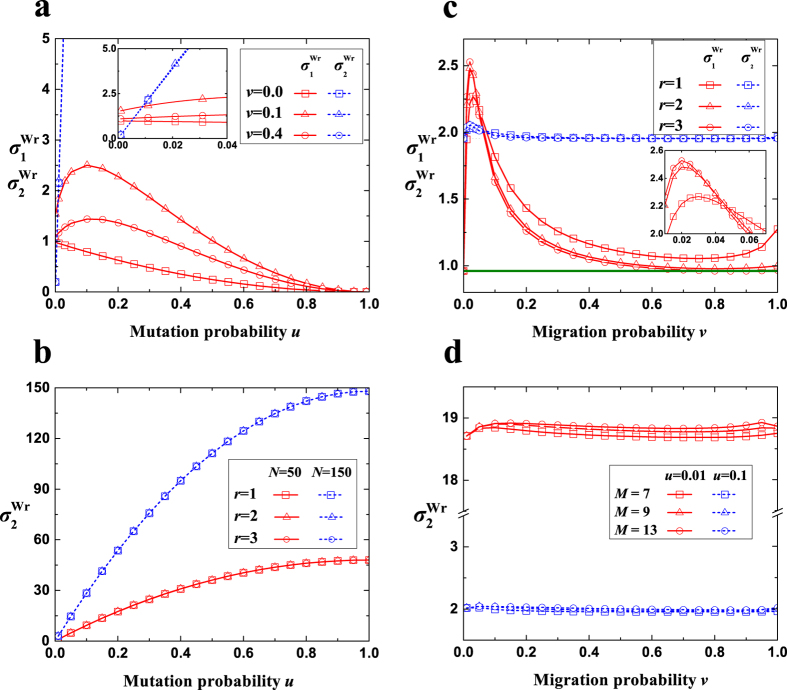
The changing trends of 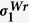 and 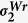. (**a**) As *u* increases, 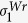 decreases for low *v* and high *v* (not shown), and exhibits an inverted U-shaped curve for moderate *v*. 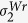 expands quickly as *u* increases. For very low *u*, 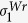 is greater than 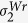 (inset). For a little higher *u*, 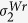 is smaller than 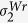, and the difference will expand quickly as *u* increases. (**b**) As *u* increases, 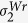 expands with a decreasing speed. The change of 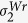 due to *r* can be neglected. (**c**) *v* or *r* leads to a significant change of 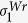 but a relatively negligible one of 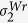. A moderate *v* near 0 maximizes 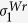, and most values of *v* near 0 result in much larger 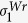 than that of the well-mixed population (solid line). There may appear a new local peak for 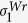 at *v* = 1. The value of *r* corresponding to the maximum value of 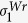 varies with *v* (inset). (**d**) 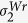 is maintained around a constant with the increase of *v* irrespective of *M*. Parameters: (**a**) *v* = 0.1, *M* = 7, *N* = 100; (**b**) *v* = 0.1, *M* = 7; (**c**) *u* = 0.01, *M* = 7, *N* = 100; (**d**) *N* = 100, *r* = 1.

Most findings of the Wright-Fisher process are qualitatively similar to those of the Moran process (Fig. 5). Compared with the well-mixed population, migration (moderate migration probabilities) causes 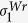 to change with the mutation probability *u* from a decreasing curve to an inverted U-shaped curve, and maintains the increasing trend of 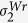 with *u*. The previous conjecture^33^ is verified that 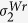 is far greater than 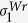 when the product of *N* (population size) and *u* is large. The mutation probabilities for 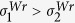 are extremely close to zero, yet those for 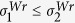 are the remaining vast majority. Migration (probability and range) results in a significant change of 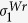 and a relatively negligible one of 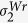. There appears a moderate migration probability maximizing 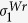, and most migration probabilities near 0 lead to much greater values of 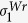 than that of the well-mixed population. The migration range corresponding to the maximum value of 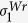 is from the longest range 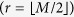 to intermediate ranges to the shortest range (*r* = 1) as the migration probability increases.

**Figure 6:**
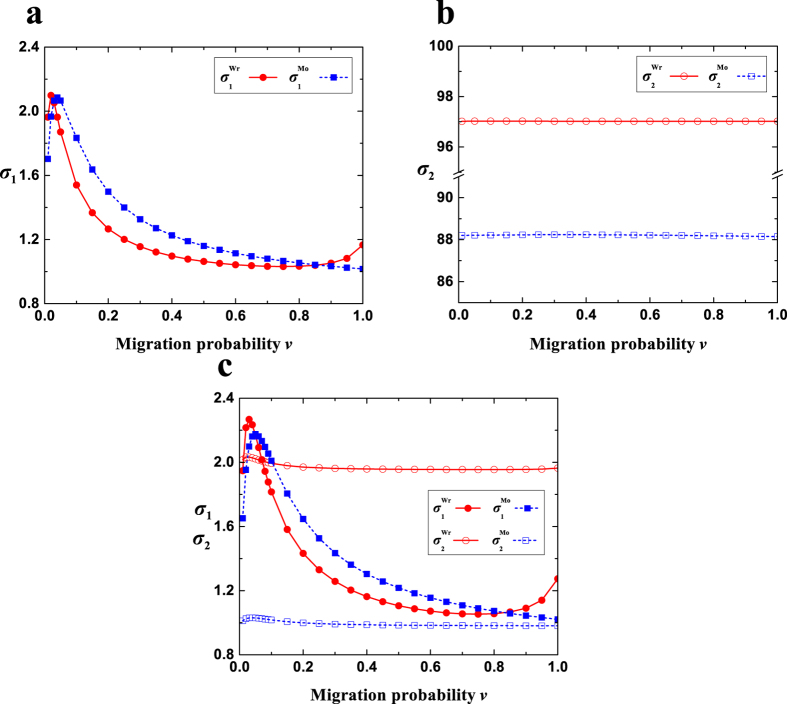
Comparison of the Wright-Fisher and the Moran process. (**a**) For extremely low *u* (*u* = 0.001), the competition of multiple strategies with equal frequency exerts a negligible influence on the multi-strategy selection (*σ*_2_ → 0), and thus we only compare 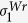 with 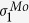. 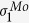 is greater than 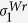 for the vast majority of *v*, and the reverse holds for the remaining few values of *v*. (**b**) For high *u* (*u* = 0.9), the pairwise competition exerts a tiny influence on the multi-strategy selection compared with the other competition (*σ*_2_ ≫ *σ*_1_), and therefore we only compare 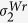 with 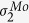. 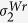 is greater than 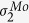 for all *v*. (**c**) For moderate *u* (*u* = 0.01), the two types of competition jointly determine the multi-strategy selection, and thus we compare the two processes based on both *σ*_1_ and *σ*_2_. The comparison of *σ*_1_ here is qualitatively similar to that of *σ*_1_ for *u* = 0.001. The comparison of *σ*_2_ here is qualitatively similar to that of *σ*_2_ for *u* = 0.9. Parameters: *N* = 100, *M* = 7, *r* = 1.

There are two qualitatively distinct ways to change *σ*_1_ and *σ*_2_ between the Moran and the Wright-Fisher process (Figs 3 and 5). The curve of 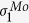 with respect to the migration probability *v* only has one peak at a low *v*; and yet in addition to this peak, there may appear a new local peak for 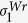 at *v* = 1. The value of 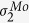 is linearly increasing with respect to the mutation probability, and yet 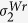 is increasing with a decreasing speed. We also quantitatively compare the two processes based on the values of *σ*_1_ and *σ*_2_ (Fig. 6). The Moran process is more effective in increasing *σ*_1_ than the Wright-Fisher process for a vast majority of migration probabilities. The Wright-Fisher process is always more effective in increasing *σ*_2_ than the Moran process, which can be seen from the value of 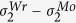 for *v* = 0 since 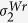 and 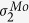 change very little with the migration probability,
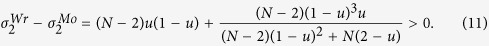


### Application: Direct reciprocity

We now use the above findings to study the evolution of cooperation under direct reciprocity. Assume that any two individuals of the same group play *m* rounds of interactions. In any one round, a cooperator brings a benefit *b* to his opponent at a cost *c* (*b* > *c* > 0), and a defector brings no benefits and pays no costs. Each individual adopts one of three strategies: *ALLC* (always cooperate) meaning one cooperates in all rounds, *ALLD* (always defect) meaning one defects in all rounds, and *TFT* (tit-for-tat) meaning one cooperates in the first round and follows his opponent’s strategy in the previous round. The payoff matrix is given by 
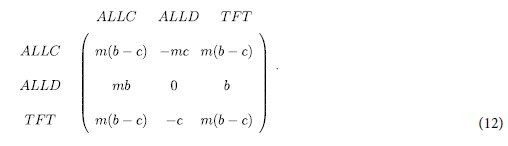


From equation (2), the condition for cooperation to be favored over defection (the average frequency of *ALLD* over the stationary distribution under weak selection, 〈*x*_*ALLD*_〉_*δ*→0_, satisfies 〈*x*_*ALLD*_〉_*δ*→0_ ≤ 1/3) is given by
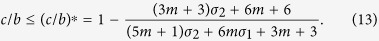


Larger critical cost-to-benefit ratio, (*c*/*b*)^*^, shows that the evolution of cooperation is favored more in the sense that more values of *c*/*b* allow natural selection to favor the evolution of cooperation.

**Figure 7:**
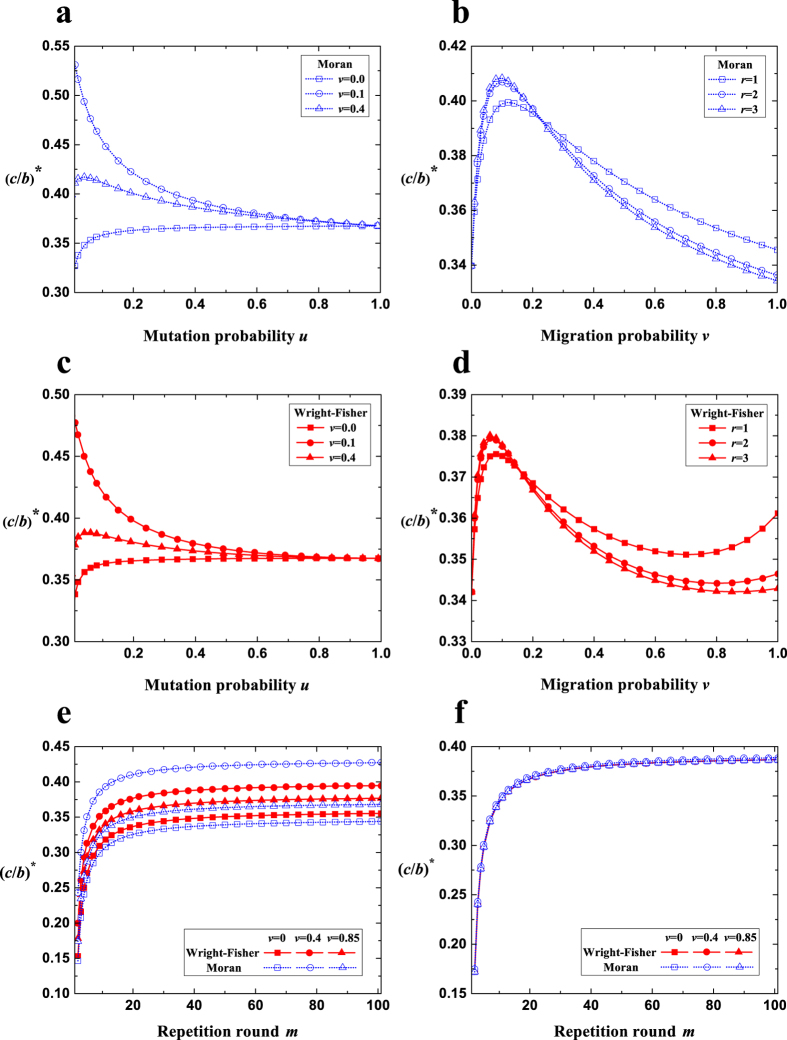
The competition of *ALLC*, *ALLD*, *TFT*. In the Moran process (**a**) and the Wright-Fisher process (**c**), (c/b)^*^ changes very little with *u* when *u* is large, but changes a lot with *u* when *u* is small. For small *u*, the changing trend of (c/b)^*^ with *u* varies with *v*. In the Moran process (**b**) and the Wright-Fisher process (**d**), the effects of *v* and *r* on (c/b)^*^ are separately similar to their effects on *σ*_1_. In the two processes (**e**,**f**) (c/b)^*^ increases with *m*. For low *u* = 0.01 (**e**), the Wright-Fisher process leads to greater (c/b)^*^ than the Moran process for very low *v* or very high *v*, and the reverse holds for moderate *v*. For high *u* = 0.9 (**f**), the two processes result in almost identical (c/b)^*^. Parameters: *N* = 100, *M* = 7, *r* = 1.

The effects of the mutation probability (*u*), the migration probability (*v*), the migration range (*r*), and the repetition round (*m*) on (*c*/*b*)^*^ are qualitatively similar but quantitatively different for the Moran and the Wright-Fisher process (Fig. 7). For large mutation probabilities, *σ*_2_ is the key determinant of (*c*/*b*)^*^ compared with *σ*_1_ since *σ*_2_ ≫ *σ*_1_. Here, (*c*/*b*)^*^ (around 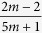) does not change much with *u* (large *u* in Fig. 7a,c), and it is almost identical for the Moran and the Wright-Fisher process irrespective of *m* and *v* (Fig. 7f). For small mutation probabilities, *σ*_1_ and *σ*_2_ jointly determine (*c*/*b*)^*^ since *σ*_1_ has a similar size to *σ*_2,_ and *σ*_1_ is the major determinant. Here, (*c*/*b*)^*^ changes a lot with *u* (low *u*) and its changing trend with respect to *u* varies with *v* (Fig. 7a,c): When the migration probability *v* is small or large, (*c*/*b*)^*^ increases with *u*, because *σ*_1_ becomes smaller (decreasing the dependence of the multi-strategy selection on the pairwise competition enhances the evolution of cooperation); When *v* is moderate, the change of (*c*/*b*)^*^ with *u* is roughly decreasing with the increase of *σ*_1_. Meanwhile for small mutation probabilities (Fig. 7e), the Wright-Fisher process leads to greater values of (*c*/*b*)^*^ than the Moran process for small and large migration probabilities satisfying 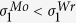, and the reverse holds for the remaining majority of migration probabilities satisfying 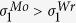. Migration (probability and range) changes *σ*_2_ very little and thus the effect of migration on (*c*/*b*)^*^ is similar to its effect on *σ*_1_ (Fig. 7b,d): There exists a moderate migration probability maximizing (*c*/*b*)^*^; The optimal migration range corresponding to the maximum value of (*c*/*b*)^*^ is from the longest to the shortest range as *v* increases (the advantage of the optimal intermediate range over other ranges is negligible). Larger repetition round (*m*) increases (*c*/*b*)^*^ by enhancing the inherent payoff advantage of *TFT* and *ALLC* over *ALLD* (Fig. 7e,f).

## Discussion

It has been proved that the strategy selection under weak selection can be expressed by several parameters (including one) independent of the payoff matrix not only for two-person games^32^,^33^ but also for multi-person games^49^. These parameters play a vital role in determining the strategy selection, but are difficult to calculate for general models. For only a few particular models, they have been obtained for two-person and two-strategy games^47^,^50^, two-person and multi-strategy games^33^, and multi-person and two-strategy games^51^,^52^. In this paper, we have focused on two-person and multi-strategy games whose strategy selection can be expressed by two parameters *σ*_1_ and *σ*_2_. We have calculated the accurate values of *σ*_1_ and *σ*_2_ for the Moran and the Wright-Fisher process in group-structured populations of any finite sizes. In a previous study^33^, the values of *σ*_1_ and *σ*_2_ have been given for large populations. The assumption of large population size guarantees that the calculation procedures are the same for the Moran and the Wright-Fisher process when *Nu* in the former and 2*Nu* in the latter are denoted by the same symbol. In finite populations, however, the calculations of *σ*_1_ and *σ*_2_ vary a lot between the two processes. Accordingly, we have separately provided their concrete calculation procedures in Supplementary Information. The key point in the two procedures is how to obtain some special probabilities under neutral selection. The special probabilities of the Moran process have been given by the previous research^31^ to investigate the evolution of cooperation in populations with two layers of group structure (whose strategy selection cannot be expressed by *σ*_1_ and *σ*_2_). The special probabilities of the Wright-Fisher process are rigorously obtained for the first time in this work.

The values of *σ*_1_ and *σ*_2_ we have calculated are appropriate for any migration patterns, mutation probabilities, migration probabilities, population sizes, and group numbers, and have been verified by Monte Carlo simulations. In previous studies^33^,^48^, the values of *σ*_1_ and *σ*_2_ have been obtained for large populations. A recent study^47^ has suggested the population size suitable for these studies^33^,^48^ is related to the mutation probability, the migration probability, and the group number. Our studies can produce their results with the assumptions of ‘global migration’ and large populations. We also have verified the previous conjecture^33^ that *σ*_2_ is far larger than *σ*_1_ when the product of the population size and the mutation probability is large. Moreover, we have obtained some new findings for the Moran and the Wright-Fisher process. Compared with the well-mixed population, migration modifies the relationship between *σ*_1_ and the mutation probability from a monotonically decreasing curve to an inverted U-shaped curve, and maintains the increasing relationship between *σ*_2_ and the mutation probability. The mutation probabilities for *σ*_1_ > *σ*_2_ (the pairwise competition dominates the multi-strategy selection) are extremely close to zero, and yet those for *σ*_1_ ≤ *σ*_2_ (the competition of multiple strategies with equal frequency dominates the multi-strategy selection) are the remaining vast majority.

We have studied how migration (probability and range) affects *σ*_1_ and *σ*_2_, and the following findings hold for the Moran and the Wright-Fisher process. Migration leads to a significant change of *σ*_1_ and a relatively negligible one of *σ*_2_. There exists a moderate migration probability maximizing *σ*_1_. The migration range leading to the largest value of *σ*_1_ decreases as *v* increases. Prior research^47^ has studied two-person and two-strategy games whose strategy selection can be expressed by a single parameter *σ*, and has analyzed the effect of migration on *σ* for the Moran process. The way that migration varies *σ*_1_ (of the multi-strategy selection) is similar to how it changes *σ* (of the two-strategy selection). This is understandable from the known equality^33^, 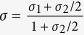, because migration can barely change *σ*_2_. Moreover, based on 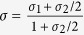, our results can be used to obtain *σ* of the Wight-Fisher process and further analyze the influence of migration on *σ*. Our results are independent of the payoff matrix and can be applied to any concrete game with multiple strategies. In particular, we have determined how migration affects the evolution of cooperation under direct reciprocity by considering the competition of *ALLC*, *ALLD*, *TFT*.

Besides games on islands^30^ and games in group-structured populations^31^, games on graphs (a single node only holds one individual at most and individuals can only migrate to empty nodes) have also been used to find what kind of migration can promote the evolution of cooperation^53^,^54^,^55^,^56^ and maintain the biological diversity^57^,^58^. Migration can be envisaged as a way to generate the coevolution of the population structure and individuals’ strategies. Compared with conventional co-evolutionary rules^59^,^60^,^61^, migration here is a very simple rule in which individuals do not have to know any information about the environment, but moderate migration (probabilities) can promote the evolution of cooperation efficiently. This suggests that moderate change of the interactive structure may be the key factor for the evolution of cooperation in the co-evolutionary dynamics.

Nearly all previous studies have only focused on either the Moran or the Wright-Fisher process^28^,^29^,^30^,^31^,^47^,^48^. Here, we have analyzed the distinct roles of the two processes in the multi-strategy selection. The curve of *σ*_1_ with respect to the migration probability *v* has only one peak at a moderate *v* in the Moran process; yet in addition to this peak, it can have a new local peak at *v* = 1 in the Wright-Fisher process. The value of *σ*_2_ is almost linearly increasing as the mutation probability expands in the Moran process, and yet it is increasing with a decreasing speed in the Wright-Fisher process. The Moran process is more effective in increasing *σ*_1_ for the vast majority of migration probabilities and the Wright-Fisher process is always more effective in increasing *σ*_2_.

Our model is a variant or a special case of previous models^28^,^29^,^30^, and our calculations of *σ*_1_ and *σ*_2_ can be extended to the multi-strategy case of these models after proper modifications. In contrast to the stepping-stone model^1^,^2^,^3^,^4^,^5^, our model requires that all subpopulations have changeable sizes instead of a fixed and equal size. Our assumption is more realistic and can bring about more interesting phenomena by introducing the typical feature of group-structured populations, ‘the asynchrony in local extinction and recolonization’. Our investigation may provide some insights into the extension of the stepping-stone models. In turn, the newest developments about the stepping-stone models may give us some ideas for analytically studying more complex and realistic models.

## Methods

By using the Mutation-Selection analysis (see Supplementary Information: I for the detailed calculations), natural selection favors the evolution of strategy *k* under weak selection (i.e., the average frequency of strategy *k* over the stationary distribution under weak selection, 〈*x*_*k*_〉_*δ*→0_, is greater than 1/*S*) if


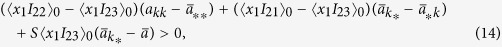


where *I*_*ij*_ is the total number of games that individuals using strategy *i* play with individuals using strategy *j* (each game played by two individuals using strategy *i* is counted twice in computing *I*_*ii*_). Comparing equation (14) with equation (2), we have the general expressions of *σ*_1_ and *σ*_2_ as


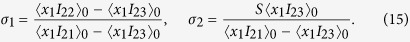


This equation gives us a simple numerical algorithm, in which *x*_1_*I*_22_ − *x*_1_*I*_23_, *Sx*_1_*I*_23_, *x*_1_*I*_21_ − *x*_1_*I*_23_ of all steady states are added up to obtain the numerators and the denominators, to perform Monte Carlo simulations.

In our model, the strategy of an individual (say *i*) is denoted by *s*_*i*_ (∈{1, 2, …, *S*}) and his location is indicated by an *M*-dimensional vector *h*_*i*_ whose *k*_*th*_ entry is 1 if he is in the *k*_*th*_ group and 0 otherwise. Each term in equation (15) can be expressed by the probabilities under neutral selection (*δ* = 0) assigned to the event that three randomly chosen (without replacement) individuals use given strategies and locations (see Supplementary Information: II for the detailed calculations),
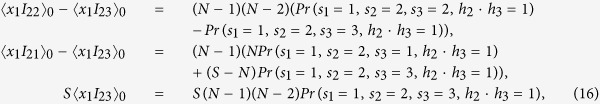
where *Pr*(*s*_1_ = *δ*_1_, *s*_2_ = *δ*_2_, *s*_3_ = *δ*_3_, *h*_2_ · *h*_3_ = 1) is the probability that three randomly chosen (without replacement) individuals (say 1, 2, 3) satisfy *s*_1_ = *δ*_1_, *s*_2_ = *δ*_2_, *s*_3_ = *δ*_3_, *h*_2_ · *h*_3_ = 1.

Note that the equations in ‘Methods’ are appropriate for the Moran and the Wright-Fisher process. The key point in calculating *σ*_1_ and *σ*_2_ is to obtain *Pr*(*s*_1_ = *δ*_1_, *s*_2_ = *δ*_2_, *s*_3_ = *δ*_3_, *h*_2_ · *h*_3_ = 1).

## Additional Information

**How to cite this article**: Zhang, Y. *et al*. Impact of migration on the multi-strategy selection in finite group-structured populations. *Sci. Rep.* **6**, 35114; doi: 10.1038/srep35114 (2016).

## Supplementary Material

Supplementary Information
