# Supplementary material for: Impact of migration on the multi-strategy selection in finite group-structured populations
Source: Sci Rep. 2016 Oct 21;6:35114. doi: 10.1038/srep35114 (PMC5073348; doi:10.1038/srep35114)
Supplement: Supplementary Information [file srep35114-s1.pdf]

Supplementary Information: Impact of migration on the multi-strategy selection in finite group-structured populations

Yanling Zhang[1], Aizhi Liu[1], Changyin Sun[2]

[1] School of Automation and Electrical Engineering, University of Science and Technology Beijing, Beijing 100083, China

[2] School of Automation, Southeast University, Nanjing 210096, China

Contents

I. The condition for $\langle x_k\rangle_{\delta\to 0} > 1/S$. 1

II. Transformations of $\langle x_1 I_{22}\rangle_0$, $\langle x_1 I_{21}\rangle_0$, and $\langle x_1 I_{23}\rangle_0$. 3

III. The calculation of $Pr(s_1 = \delta_1, s_2 = \delta_2, s_3 = \delta_3, h_2 \cdot h_3 = 1)$ for 'global mutation' when the Moran process is used. 5

IV. The calculation of $Pr(s_1 = \delta_1, s_2 = \delta_2, s_3 = \delta_3, h_2 \cdot h_3 = 1)$ for any mutation pattern when the Wright-Fisher process is used. 10

V. The calculation of $Pr(s_1 = \delta_1, s_2 = \delta_2, s_3 = \delta_3, h_2 \cdot h_3 = 1)$ for 'global mutation' when the Wright-Fisher process is used. 15

VI. Migration occurs before reproduction. 16

References 17

I. THE CONDITION FOR $\langle x_k\rangle_{\delta\to 0} > 1/S$.

For the Moran process, the mean frequency of strategy k averaged over the stationary distribution is calculated by the Mutation-Selection analysis as follows. In a single-step update, the frequency of strategy k denoted by x_k is increased in two ways: one existing individual using s-trategy k reproduces, and the offspring does not mutate to other strategies; one existing individual

using other strategies reproduces, and the offspring mutates to strategy k. Yet in a single-step update, there is only one way to decrease x_k: one existing individual using strategy k dies. Thus, the expected change of x_k in a single-step update is $\triangle x_k = (1-\frac{(S-1)u}{S})\frac{F_k}{F} + \frac{u}{S}(1-\frac{F_k}{F}) - x_k$, where F_k and F are the total fitness of individuals using strategy k and of the population, respectively. Since the mean $\triangle x_k$ averaged over the stationary distribution is zero, then $\langle x_k\rangle_\delta = \frac{1}{S} + \frac{1-u}{u}\langle \frac{F_k}{F} - x_k\rangle_\delta$, where $\langle X\rangle_\delta$ represents the quantity which is averaged over all steady states weighted by the steady-state probabilities (with the selection intensity δ). Performing the perturbation theory in the limit $\delta \to 0$, we have

$$\langle x_k\rangle_{\delta\to 0} = \frac{1}{S} + \delta\frac{1-u}{Nu}\langle P_k - x_k P\rangle_0, \tag{1}$$

where $P_k = \sum_{j=1}^{S} a_{kj} I_{kj}$ and $P = \sum_{k=1}^{S}\sum_{j=1}^{S} a_{kj} I_{kj}$ are the total payoffs of individuals using strategy k and of the population, respectively. Accordingly, natural selection favors the evolution of strategy k under weak selection (i.e., $\langle x_k\rangle_{\delta\to 0} > 1/S$) if

$$\langle P_k - x_k P\rangle_0 > 0. \tag{2}$$

Although Eq. (2) aims at the Moran process, it also holds for the Wright-Fisher process, where the expected number of individuals using strategy k who are reproduced in one update step is $N((1-\frac{(S-1)u}{S})\frac{F_k}{F} + \frac{u}{S}(1-\frac{F_k}{F}))$ and the expected number of individuals using strategy k who die in one update step is Nx_k.

Assuming I_{ij} is the total number of games that individuals using strategy i play with individuals using strategy j (each game played by two individuals using strategy i is counted twice in computing I_{ii}), we write $\langle P_k - x_k P\rangle_0$ as

$$\begin{aligned}&\langle P_k - x_k P\rangle_0 = \\ &S(\langle x_1 I_{22}\rangle_0 - \langle x_1 I_{23}\rangle_0)(a_{kk} - \overline{a}_{**}) + S(\langle x_1 I_{21}\rangle_0 - \langle x_1 I_{23}\rangle_0)(\overline{a}_{k*} - \overline{a}_{*k}) + S^2\langle x_1 I_{23}\rangle_0(\overline{a}_{k*} - \overline{a}),\end{aligned} \tag{3}$$

where $\overline{a}_{**} = \frac{1}{S}\sum_{i=1}^{S} a_{ii}$, $\overline{a}_{k*} = \frac{1}{S}\sum_{i=1}^{S} a_{ki}$, $\overline{a}_{*k} = \frac{1}{S}\sum_{i=1}^{S} a_{ik}$, and $\overline{a} = \frac{1}{S^2}\sum_{i=1}^{S}\sum_{j=1}^{S} a_{ij}$. The equal sign uses the following equations

$$\langle x_1 I_{11}\rangle_0 = \langle x_p I_{pp}\rangle_0, \langle x_1 I_{12}\rangle_0 = \langle x_p I_{pq}\rangle_0, \langle x_1 I_{21}\rangle_0 = \langle x_p I_{qp}\rangle_0, \langle x_1 I_{22}\rangle_0 = \langle x_p I_{qq}\rangle_0, \langle x_1 I_{23}\rangle_0 = \langle x_p I_{qr}\rangle_0,$$

where $p \neq q \neq r \neq p$. These equations hold because all strategies in the neutral stationary state are equivalent, meaning $\langle x_p I_{qr}\rangle_0 = \langle x_{p'} I_{q'r'}\rangle_0$ when a bijection operation from the set $\{1, 2, \cdots, S\}$ to $\{1, 2, \cdots, S\}$ satisfies $\pi((p, q, r)) = (p', q', r')$.

II. TRANSFORMATIONS OF $\langle x_1 I_{22}\rangle_0$, $\langle x_1 I_{21}\rangle_0$, AND $\langle x_1 I_{23}\rangle_0$.

Assume the strategy of an individual (say i) is denoted by s_i ($\in \{1, 2, \cdots, S\}$) and his location by an M-dimensional vector h_i whose k_{th} entry is 1 if he is in the k_{th} group and 0 otherwise, and we have,

$$\begin{aligned}\langle x_1 I_{22}\rangle_0 &= \langle \textstyle\sum_{p,q,r} \mathbf{1}_{s_p=1,s_q=2,s_r=2,h_q\cdot h_r=1}\rangle_0/N - \langle \textstyle\sum_{p,q} \mathbf{1}_{s_p=1,s_q=2}\rangle_0/N,\\ \langle x_1 I_{21}\rangle_0 &= \langle \textstyle\sum_{p,q,r} \mathbf{1}_{s_p=1,s_q=2,s_r=1,h_q\cdot h_r=1}\rangle_0/N,\\ \langle x_1 I_{23}\rangle_0 &= \langle \textstyle\sum_{p,q,r} \mathbf{1}_{s_p=1,s_q=2,s_r=3,h_q\cdot h_r=1}\rangle_0/N.\end{aligned} \tag{4}$$

Next, we will show how each term on the right side is expressed by some probabilities under neutral selection ($\delta = 0$) assigned to the event that three or two randomly chosen (without replacement) individuals have given strategies and locations.

Assume the expectation E is taken over all possible triples of (l, i, j) or all possible pairs of (l, i), and we get

$$\begin{aligned}\langle \textstyle\sum_{p,q,r} \mathbf{1}_{s_p=1,s_q=2,s_r=2,h_q\cdot h_r=1}\rangle_0 &= N^3 E[\langle \mathbf{1}_{s_p=1,s_q=2,s_r=2,h_q\cdot h_r=1}\rangle_0],\\ \langle \textstyle\sum_{p,q} \mathbf{1}_{s_p=1,s_q=2}\rangle_0 &= N^2 E[\mathbf{1}_{s_p=1,s_q=2}],\\ \langle \textstyle\sum_{p,q,r} \mathbf{1}_{s_p=1,s_q=2,s_r=1,h_q\cdot h_r=1}\rangle_0 &= N^3 E[\langle \mathbf{1}_{s_p=1,s_q=2,s_r=1,h_q\cdot h_r=1}\rangle_0],\\ \langle \textstyle\sum_{p,q,r} \mathbf{1}_{s_p=1,s_q=2,s_r=3,h_q\cdot h_r=1}\rangle_0 &= N^3 E[\langle \mathbf{1}_{s_p=1,s_q=2,s_r=3,h_q\cdot h_r=1}\rangle_0].\end{aligned} \tag{5}$$

The sum over p, q, r without limitation means that three individuals are chosen randomly and with replacement from the population: with probability $1/N^2$, all the three individuals are identical ($p = q = r$); with probability $(N-1)/N^2$, two given individuals are the same but the third is different ($p = q \neq r$, $p = r \neq q$, or $q = r \neq p$); with probability $(N-1)(N-2)/N^2$, the three are different from each other ($p \neq q \neq r \neq p$). The sum over p, q without limitation means that two individuals are chosen randomly and with replacement from the population: with probability $1/N$, the two individuals are identical ($p = q$); with probability $(N-1)/N$, they are different ($p \neq q$). Therefore, we have

$$\begin{aligned}E[\langle \mathbf{1}_{s_p=1,s_q=2,s_r=2,h_q\cdot h_r=1}\rangle_0] &= \tfrac{N-1}{N^2}\langle \mathbf{1}_{s_p=1,s_q=2|p\neq q}\rangle_0 + \tfrac{(N-1)(N-2)}{N^2}\langle \mathbf{1}_{s_p=1,s_q=2,s_r=2,h_q\cdot h_r=1|p\neq q\neq r\neq p}\rangle_0,\\ E[\langle \mathbf{1}_{s_p=1,s_q=2}\rangle_0] &= \tfrac{N-1}{N}\langle \mathbf{1}_{s_p=1,s_q=2|p\neq q}\rangle_0,\\ E[\langle \mathbf{1}_{s_p=1,s_q=2,s_r=1,h_q\cdot h_r=1}\rangle_0] &= \tfrac{N-1}{N^2}\langle \mathbf{1}_{s_p=1,s_q=2,h_p\cdot h_q=1|p\neq q}\rangle_0 + \tfrac{(N-1)(N-2)}{N^2}\langle \mathbf{1}_{s_p=1,s_q=2,s_r=1,h_q\cdot h_r=1|p\neq q\neq r\neq p}\rangle_0,\\ E[\langle \mathbf{1}_{s_p=1,s_q=2,s_r=3,h_q\cdot h_r=1}\rangle_0] &= \tfrac{(N-1)(N-2)}{N^2}\langle \mathbf{1}_{s_p=1,s_q=2,s_r=3,h_q\cdot h_r=1|p\neq q\neq r\neq p}\rangle_0.\end{aligned} \tag{6}$$

Let $Pr(X)$ be the probability that X occurs, then $\langle \mathbf{1}_{s_p=1,s_q=2,s_r=2,h_q\cdot h_r=1|p\neq q\neq r\neq p}\rangle_0 = Pr(s_p = 1, s_q = 2, s_r = 2, h_q \cdot h_r = 1|p \neq q \neq r \neq p)$. $Pr(s_p = 1, s_q = 2, s_r = 2, h_q \cdot h_r = 1|p \neq q \neq r \neq p)$ can be

understood as the probability that three randomly chosen (without replacement) individuals (say $1, 2, 3$) satisfy $h_2 \cdot h_3 = 1$, $s_1 = 1$, $s_2 = 2$, $s_3 = 2$. Assume $Pr(s_1 = a, s_2 = b, s_3 = c, h_2 \cdot h_3 = 1) = Pr(s_l = a, s_i = b, s_j = c, h_i \cdot h_j = 1 | l \neq i \neq j \neq l)$ for simplicity, and then we have

$$\langle \mathbf{1}_{s_p=1, s_q=2, s_r=2, h_q \cdot h_r=1 | p\neq q\neq r\neq p} \rangle_0 = Pr(s_1 = 1, s_2 = 2, s_3 = 2, h_2 \cdot h_3 = 1). \tag{7}$$

Similarly, other terms on the right side in Eq. (6) are given by

$$\begin{aligned}
&\langle \mathbf{1}_{s_p=1, s_q=2 | p\neq q} \rangle_0 = Pr(s_1 = 1, s_2 = 2),\\
&\langle \mathbf{1}_{s_p=1, s_q=2, h_p \cdot h_q=1 | p\neq q} \rangle_0 = Pr(s_1 = 1, s_2 = 2, h_1 \cdot h_2 = 1),\\
&\langle \mathbf{1}_{s_p=1, s_q=2, s_r=1, h_q \cdot h_r=1 | p\neq q\neq r\neq p} \rangle_0 = Pr(s_1 = 1, s_2 = 2, s_3 = 1, h_2 \cdot h_3 = 1),\\
&\langle \mathbf{1}_{s_p=1, s_q=2, s_r=3, h_q \cdot h_r=1 | p\neq q\neq r\neq p} \rangle_0 = Pr(s_1 = 1, s_2 = 2, s_3 = 3, h_2 \cdot h_3 = 1).
\end{aligned} \tag{8}$$

$Pr(s_1 = a, s_2 = b) = Pr(s_i = a, s_j = b | i \neq j)$ is the probability that two randomly chosen (without replacement) individuals (say $1, 2$) satisfy $s_1 = a$, $s_2 = b$, and $Pr(s_1 = a, s_2 = b, h_1 \cdot h_2 = 1) = Pr(s_i = a, s_j = b, h_i \cdot h_j = 1 | i \neq j)$ is the probability that two randomly chosen (without replacement) individuals (say $1, 2$) satisfy $s_1 = a, s_2 = b, h_1 \cdot h_2 = 1$. Substituting Eqs. (8), (7), (6) into Eq. (5), we have

$$\begin{aligned}
&\langle \textstyle\sum_{p,q,r} \mathbf{1}_{s_p=1, s_q=2, s_r=2, h_q \cdot h_r=1} \rangle_0 = N(N-1)Pr(s_1 = 1, s_2 = 2) + N(N-1)(N-2)Pr(s_1 = 1,\\
&s_2 = 2, s_3 = 2, h_2 \cdot h_3 = 1),\\
&\langle \textstyle\sum_{p,q} \mathbf{1}_{s_p=1, s_q=2} \rangle_0 = N(N-1)Pr(s_1 = 1, s_2 = 2),\\
&\langle \textstyle\sum_{p,q,r} \mathbf{1}_{s_p=1, s_q=2, s_r=1, h_q \cdot h_r=1} \rangle_0 = N(N-1)Pr(s_1 = 1, s_2 = 2, h_1 \cdot h_2 = 1) + N(N-1)\\
&(N-2)Pr(s_1 = 1, s_2 = 2, s_3 = 1, h_2 \cdot h_3 = 1),\\
&\langle \textstyle\sum_{p,q,r} \mathbf{1}_{s_p=1, s_q=2, s_r=3, h_q \cdot h_r=1} \rangle_0 = N(N-1)(N-2)Pr(s_1 = 1, s_2 = 2, s_3 = 3, h_2 \cdot h_3 = 1).
\end{aligned} \tag{9}$$

From Eqs. (9), (4), we have

$$\begin{aligned}
&\langle x_1 I_{22} \rangle_0 = (N-1)(N-2)Pr(s_1 = 1, s_2 = 2, s_3 = 2, h_2 \cdot h_3 = 1),\\
&\langle x_1 I_{21} \rangle_0 = (N-1)Pr(s_1 = 1, s_2 = 2, h_1 \cdot h_2 = 1) + (N-1)\\
&(N-2)Pr(s_1 = 1, s_2 = 2, s_3 = 1, h_2 \cdot h_3 = 1),\\
&\langle x_1 I_{23} \rangle_0 = (N-1)(N-2)Pr(s_1 = 1, s_2 = 2, s_3 = 3, h_2 \cdot h_3 = 1).
\end{aligned} \tag{10}$$

III. THE CALCULATION OF $Pr(s_1 = \delta_1, s_2 = \delta_2, s_3 = \delta_3, h_2 \cdot h_3 = 1)$ FOR 'GLOBAL MUTATION' WHEN THE MORAN PROCESS IS USED.

In previous research [1], the general expression of $Pr(s_1 = \delta_1, s_2 = \delta_2, s_3 = \delta_3, h_2 \cdot h_3 = 1)$ has been given by

$$Pr(s_1 = \delta_1, s_2 = \delta_2, s_3 = \delta_3, h_2 \cdot h_3 = 1) = \frac{1}{3MS^3} \sum_{z_1=M, z_2+z_3=M or 2M} \sum_{w_1+w_2+w_3=S,2S or 3S} (\sum_{x_1=z_1+z_2, x_2=z_3, y_1=w_1+w_2, y_2=w_3} + \sum_{x_1=z_1+z_3, x_2=z_2, y_1=w_1+w_3, y_2=w_2} + \sum_{x_1=z_2+z_3, x_2=z_1, y_1=w_2+w_3, y_2=w_1})\Phi(f(z_1), f(z_2), f(z_3), g(w_1), g(w_2), g(w_3))\Psi(f(x_1), f(x_2), g(y_1), g(y_2)) \exp(-\frac{2\pi i}{S}(w_1 \cdot \delta_1 + w_2 \cdot \delta_2 + w_3 \cdot \delta_3)), \tag{11}$$

where

$$\Psi(f(x_1), f(x_2), g(y_1), g(y_2)) = \frac{(1-u)(1-v)+(1-u)v\sum_{i=1}^2 f(x_i)/2+(1-v)u\sum_{i=1}^2 g(y_i)/2+uv\sum_{i=1}^2 f(x_i)g(y_i)/2}{1+(N-1)(1-u)v(1-\sum_{i=1}^2 \frac{f(x_i)}{2})+(N-1)(1-v)u(1-\sum_{i=1}^2 \frac{g(y_i)}{2})+(N-1)uv(1-\sum_{i=1}^2 \frac{f(x_i)g(y_i)}{2})}, \tag{12}$$

$$\Phi(f(z_1), f(z_2), f(z_3), g(w_1), g(w_2), g(w_3)) = \begin{cases} \frac{2(1-u)(1-v)+(1-u)v(f(z_1)+f(z_2))+(1-v)u(g(w_1)+g(w_2))+uv(f(z_1)g(w_1)+f(z_2)g(w_2))}{2+(N-2)(1-u)v(1-\sum_{i=1}^3 \frac{f(z_i)}{3})+(N-2)(1-v)u(1-\sum_{i=1}^3 \frac{g(w_i)}{3})+(N-2)uv(1-\sum_{i=1}^3 \frac{f(z_i)g(w_i)}{3})}, \\ \text{if } x_1 = z_1 + z_2, x_2 = z_3, y_1 = w_1 + w_2, y_2 = w_3, \\ \frac{2(1-u)(1-v)+(1-u)v(f(z_1)+f(z_3))+(1-v)u(g(w_1)+g(w_3))+uv(f(z_1)g(w_1)+f(z_3)g(w_3))}{2+(N-2)(1-u)v(1-\sum_{i=1}^3 \frac{f(z_i)}{3})+(N-2)(1-v)u(1-\sum_{i=1}^3 \frac{g(w_i)}{3})+(N-2)uv(1-\sum_{i=1}^3 \frac{f(z_i)g(w_i)}{3})}, \\ \text{if } x_1 = z_1 + z_3, x_2 = z_2, y_1 = w_1 + w_3, y_2 = w_2, \\ \frac{2(1-u)(1-v)+(1-u)v(f(z_2)+f(z_3))+(1-v)u(g(w_2)+g(w_3))+uv(f(z_2)g(w_2)+f(z_3)g(w_3))}{2+(N-2)(1-u)v(1-\sum_{i=1}^3 \frac{f(z_i)}{3})+(N-2)(1-v)u(1-\sum_{i=1}^3 \frac{g(w_i)}{3})+(N-2)uv(1-\sum_{i=1}^3 \frac{f(z_i)g(w_i)}{3})}, \\ \text{if } x_1 = z_2 + z_3, x_2 = z_1, y_1 = w_2 + w_3, y_2 = w_1. \end{cases} \tag{13}$$

$g(x)$ ($f(x)$) corresponds to the structure function of the random walk describing the mutation process (the migration process) along a lineage, and satisfies $g(S) = 1$ and $g(x) = g(S - x)$ ($f(M) = 1$ and $f(x) = f(M - x)$). Note that w_1, w_2, w_3, y_1, y_2 (z_1, z_2, z_3, x_1, x_2) can take on only integers between 1 and S (M) including the boundary. The calculation procedure is divided into two steps. In step one, from the present (the time when three individuals are randomly chosen from the population without replacement) backwards to the time of their most recent common ancestor (MRCA), the coalescence theory is used to acquire the distribution about the number of migration events and of mutation events along each lineage in the ancestral process. Here, there are three possible pairs for three individuals (say $1, 2, 3$) to first coalesce into a common ancestor: 1 and 2 (corresponding to $x_1 = z_1 + z_2, x_2 = z_3, y_1 = w_1 + w_2, y_2 = w_3$), 1 and 3 (corresponding to $x_1 = z_1 + z_3, x_2 = z_2, y_1 = w_1 + w_3, y_2 = w_2$), 2 and 3 (corresponding to $x_1 = z_2 + z_3, x_2 = z_1, y_1 = w_2 + w_3, y_2 = w_1$). In step two, from the time of MRCA forwards to the present, the random walk is employed to trace the changing path of

the strategy and of the location along each lineage. Here, six random walkers (corresponding to $g(w_1), g(w_2), g(w_3), f(z_1), f(z_2), f(z_3)$) record the strategies and the locations of three individuals' ancestors before their first coalescence, and then four random walkers (corresponding to $g(y_1), g(y_2), f(x_1), f(x_2)$) record the strategies and the locations of the three individuals' ancestors between their first coalescence and their second coalescence.

In our model, the offspring will adopt one of S ($S > 2$) strategies once a mutation occurs. Therefore, we have $g(x) = \frac{1}{S}(1 + \cos(\frac{2\pi x}{S}) + \cdots + \cos(\frac{2\pi(S-1)x}{S}))$. According to the values of $g(w_1)$, $g(w_2)$, $g(w_3)$, there is no need to divide the sets $A_1 = \{(w_1, w_2, w_3)| \sum_{x=1}^{3} w_x = 3S\}$ and $A_2 = \{(w_1, w_2, w_3)| \sum_{x=1}^{3} w_x = S\}$, but the set $\{(w_1, w_2, w_3)| \sum_{x=1}^{3} w_x = 2S\}$ can further be divided into four sets $A_3 = \{(w_1, w_2, w_3)|w_1, w_2, w_3 \neq S, \sum_{x=1}^{3} w_x = 2S\}$, $A_4 = \{(w_1, w_2, w_3)|w_1 = S, \sum_{x=1}^{3} w_x = 2S\}$, $A_5 = \{(w_1, w_2, w_3)|w_2 = S, \sum_{x=1}^{3} w_x = 2S\}$, $A_6 = \{(w_1, w_2, w_3)|w_3 = S, \sum_{x=1}^{3} w_x = 2S\}$. The values of $g(w_1)$, $g(w_2)$ and $g(w_3)$ over these sets are as follows,

$$\begin{cases} g(w_1) = 1, g(w_2) = 1, g(w_3) = 1, & \text{if } (w_1, w_2, w_3) \in A_1; \\ g(w_1) = 0, g(w_2) = 0, g(w_3) = 0, & \text{if } (w_1, w_2, w_3) \in A_2 \cup A_3; \\ g(w_1) = 1, g(w_2) = 0, g(w_3) = 0, & \text{if } (w_1, w_2, w_3) \in A_4; \\ g(w_1) = 0, g(w_2) = 1, g(w_3) = 0, & \text{if } (w_1, w_2, w_3) \in A_5; \\ g(w_1) = 0, g(w_2) = 0, g(w_3) = 1, & \text{if } (w_1, w_2, w_3) \in A_6. \end{cases} \tag{14}$$

Meanwhile, the set $\{(z_1, z_2, z_3)|z_1 = M, z_2 + z_3 = M \text{ or } 2M\}$ satisfies

$$f(z_1) = 1, \quad f(z_2) = f(z_3). \tag{15}$$

In the case of $\{x_1 = z_1 + z_2, x_2 = z_3, y_1 = w_1 + w_2, y_2 = w_3\}$, we have

$$\begin{cases} g(y_1) = 1, \quad g(y_2) = 1, & \text{if } (w_1, w_2, w_3) \in A_1 \cup A_6; \\ g(y_1) = 0, \quad g(y_2) = 0, & \text{if } (w_1, w_2, w_3) \in A_2 \cup A_3 \cup A_4 \cup A_5; \\ f(x_1) = f(x_2) = f(z_2), & \text{for } z_1 = M, z_2 + z_3 = M \text{ or } 2M. \end{cases} \tag{16}$$

Substituting Eqs. (14), (15) into Eq. (13) (the sign $\cdots$ in $\Phi(\cdots)$ means $f(z_1), f(z_2), f(z_3), g(w_1)$,

$g(w_2), g(w_3))$,

$$\Phi(\cdots) = \begin{cases} L_1(f(z_2)) = \frac{2-v+vf(z_2)}{2+\frac{2(N-2)v}{3}(1-f(z_2))}, & \text{if } (w_1,w_2,w_3) \in A_1; \\ \Phi_1(f(z_2)) = \frac{(1-u)(2-v+vf(z_2))}{2+(N-2)u+\frac{2(N-2)(1-u)v}{3}(1-f(z_2))}, & \text{if } (w_1,w_2,w_3) \in A_2 \cup A_3; \\ L_2(f(z_2)) = \frac{1+(1-u)(1-v+vf(z_2))}{2+\frac{2(N-2)u}{3}+\frac{2(N-2)(1-u)v}{3}(1-f(z_2))}, & \text{if } (w_1,w_2,w_3) \in A_4; \\ \Phi_2(f(z_2)) = \frac{2-u-v+vf(z_2)}{2+\frac{2(N-2)u}{3}+\frac{(N-2)(2-u)v}{3}(1-f(z_2))}, & \text{if } (w_1,w_2,w_3) \in A_5; \\ \Phi_3(f(z_2)) = \frac{(1-u)(2-v+vf(z_2))}{2+\frac{2(N-2)u}{3}+\frac{(N-2)(2-u)v}{3}(1-f(z_2))}, & \text{if } (w_1,w_2,w_3) \in A_6. \end{cases} \tag{17}$$

Substituting Eq. (16) into Eq. (12) (the sign $\cdots$ in $\Psi(\cdots)$ means $f(x_1), f(x_2), g(y_1), g(y_2)$), we have

$$\Psi(\cdots) = \begin{cases} \Psi_1(f(z_2)) = \frac{1-v+vf(z_2)}{1+(N-1)v(1-f(z_2))}, & \text{if } (w_1,w_2,w_3) \in A_1 \cup A_6; \\ \Psi_2(f(z_2)) = \frac{(1-u)(1-v+vf(z_2))}{1+(N-1)u+(N-1)(1-u)v(1-f(z_2))}, & \text{if } (w_1,w_2,w_3) \in A_2 \cup A_3 \cup A_4 \cup A_5. \end{cases} \tag{18}$$

In the case of $\{x_1 = z_1 + z_3, x_2 = z_2, y_1 = w_1 + w_3, y_2 = w_2\}$, we have

$$\begin{cases} g(y_1) = 1, \quad g(y_2) = 1, & \text{if } (w_1,w_2,w_3) \in A_1 \cup A_5; \\ g(y_1) = 0, \quad g(y_2) = 0, & \text{if } (w_1,w_2,w_3) \in A_2 \cup A_3 \cup A_4 \cup A_6; \\ f(x_1) = f(x_2) = f(z_2), & \text{for } z_1 = M, z_2 + z_3 = M \text{ or } 2M. \end{cases} \tag{19}$$

Substituting Eqs. (14), (15) into Eq.(13), we have

$$\Phi(\cdots) = \begin{cases} L_1(f(z_2)), & \text{if } (w_1,w_2,w_3) \in A_1. \\ \Phi_1(f(z_2)), & \text{if } (w_1,w_2,w_3) \in A_2 \cup A_3; \\ L_2(f(z_2)), & \text{if } (w_1,w_2,w_3) \in A_4; \\ \Phi_3(f(z_2)), & \text{if } (w_1,w_2,w_3) \in A_5; \\ \Phi_2(f(z_2)), & \text{if } (w_1,w_2,w_3) \in A_6. \end{cases} \tag{20}$$

Substituting Eq. (19) into Eq. (12), we have

$$\Psi(\cdots) = \begin{cases} \Psi_1(f(z_2)), & \text{if } (w_1,w_2,w_3) \in A_1 \cup A_5; \\ \Psi_2(f(z_2)), & \text{if } (w_1,w_2,w_3) \in A_2 \cup A_3 \cup A_4 \cup A_6. \end{cases} \tag{21}$$

In the case of $\{x_1 = z_2 + z_3, x_2 = z_1, y_1 = w_2 + w_3, y_2 = w_1\}$, we have

$$\begin{cases} g(y_1) = 1, \quad g(y_2) = 1, & \text{if } (w_1,w_2,w_3) \in A_1 \cup A_4; \\ g(y_1) = 0, \quad g(y_2) = 0, & \text{if } (w_1,w_2,w_3) \in A_2 \cup A_3 \cup A_5 \cup A_6; \\ f(x_1) = 1, \quad f(x_2) = 1, & \text{for } z_1 = M, z_2 + z_3 = M \text{ or } 2M. \end{cases} \tag{22}$$

Substituting Eqs. (14), (15) into Eq.(13), we have

$$\Phi(\cdots)=\begin{cases} L_3(f(z_2))=\frac{1-v+vf(z_2)}{1+\frac{(N-2)v}{3}(1-f(z_2))}, & \text{if } (w_1,w_2,w_3)\in A_1;\\ \Phi_4(f(z_2))=\frac{(1-u)(1-v+vf(z_2))}{1+\frac{(N-2)u}{2}+\frac{(N-2)(1-u)v}{3}(1-f(z_2))}, & \text{if } (w_1,w_2,w_3)\in A_2\cup A_3;\\ L_4(f(z_2))=\frac{(1-u)(1-v+vf(z_2))}{1+\frac{(N-2)u}{3}+\frac{(N-2)(1-u)v}{3}(1-f(z_2))}, & \text{if } (w_1,w_2,w_3)\in A_4;\\ \Phi_5(f(z_2))=\frac{(2-u)(1-v+vf(z_2))}{2+\frac{2(N-2)u}{3}+\frac{(N-2)(2-u)v}{3}(1-f(z_2))}, & \text{if } (w_1,w_2,w_3)\in A_5;\\ \Phi_5(f(z_2)), & \text{if } (w_1,w_2,w_3)\in A_6. \end{cases} \tag{23}$$

Substituting Eq. (22) into Eq. (12), we have

$$\Psi(\cdots)=\begin{cases} 1, & \text{if } (w_1,w_2,w_3)\in A_1\cup A_4;\\ \alpha_1=\frac{1-u}{1+(N-1)u}, & \text{if } (w_1,w_2,w_3)\in A_2\cup A_3\cup A_5\cup A_6. \end{cases} \tag{24}$$

According to Eqs. (17), (18), (20), (21), (23), (24), the expressions of $\Phi(\cdots)\Psi(\cdots)$ for the point $(w_1,w_2,w_3)\in(\cup_{x=1}^{6}A_x)$ and different coalescence combinations are summarized in TABLE I.

TABLE I: The expressions of $\Phi(\cdots)\Psi(\cdots)$ for the point $(w_1,w_2,w_3)\in(\cup_{x=1}^{6}A_x)$ and different coalescence combinations (the sign $\cdot$ in the table means $f(z_2)$) .

	$x_1=z_1+z_2, x_2=z_3$ $y_1=w_1+w_2, y_2=w_3$	$x_1=z_1+z_3, x_2=z_2$ $y_1=w_1+w_3, y_2=w_2$	$x_1=z_2+z_3, x_2=z_1$ $y_1=w_2+w_3, y_2=w_1$
$(w_1,w_2,w_3)\in A_1$	$L_1(\cdot)\Psi_1(\cdot)$	$L_1(\cdot)\Psi_1(\cdot)$	$L_3(\cdot)$
$(w_1,w_2,w_3)\in(A_2\cup A_3)$	$\Phi_1(\cdot)\Psi_2(\cdot)$	$\Phi_1(\cdot)\Psi_2(\cdot)$	$\Phi_4(\cdot)\alpha_1$
$(w_1,w_2,w_3)\in A_4$	$L_2(\cdot)\Psi_2(\cdot)$	$L_2(\cdot)\Psi_2(\cdot)$	$L_4(\cdot)$
$(w_1,w_2,w_3)\in A_5$	$\Phi_2(\cdot)\Psi_2(\cdot)$	$\Phi_3(\cdot)\Psi_1(\cdot)$	$\Phi_5(\cdot)\alpha_1$
$(w_1,w_2,w_3)\in A_6$	$\Phi_3(\cdot)\Psi_1(\cdot)$	$\Phi_2(\cdot)\Psi_2(\cdot)$	$\Phi_5(\cdot)\alpha_1$

The set A_2 is equivalent to the set $\{(w_1,w_2,w_3)|w_1\in\{1,2,\cdots,S-2\}, w_2\in\{1,2,\cdots,S-1-w_1\}, w_3=S-w_1-w_2\}$. The set A_3 is equivalent to the set $\{(w_1,w_2,w_3)|w_1\in\{2,\cdots,S-1\}, w_2\in$

$\{S - w_1 + 1, \cdots, S - 1\}, w_3 = 2S - w_1 - w_2\}$. Therefore, we have

$$\sum_{(w_1,w_2,w_3)\in\{A_2\cup A_3\}} \exp\{-\frac{2\pi i}{S}(w_1 \cdot \delta_1 + w_2 \cdot \delta_2 + w_3 \cdot \delta_3)\}$$
$$= \sum_{w_1=1}^{S-1}\{\sum_{w_2=1}^{S-w_1-1} + \sum_{w_2=S-w_1+1}^{S-1}\}\exp\{-\frac{2\pi i}{S}[(\delta_1 - \delta_3)w_1 + (\delta_2 - \delta_3)w_2]\}$$
$$= \begin{cases} \sum_{w_1=1}^{S-1} \exp\{-\frac{2\pi i}{S}(\delta_1 - \delta_3)w_1\}(-1 - \exp\{\frac{2\pi i}{S}(\delta_2 - \delta_3)w_1\}), & \text{if } \delta_2 \neq \delta_3; \\ \sum_{w_1=1}^{S-1} \exp\{-\frac{2\pi i}{S}(\delta_1 - \delta_3)w_1\}(S - 2), & \text{if } \delta_2 = \delta_3; \end{cases}$$
$$= \begin{cases} 2, & \text{if } \delta_1 \neq \delta_2 \neq \delta_3 \neq \delta_1; \\ -S + 2, & \text{if } \delta_1 = \delta_2 \neq \delta_3; \\ -S + 2, & \text{if } \delta_1 = \delta_3 \neq \delta_2; \\ -S + 2, & \text{if } \delta_2 = \delta_3 \neq \delta_1; \\ (S - 1)(S - 2), & \text{if } \delta_1 = \delta_2 = \delta_3. \end{cases}$$

Similarly, we can separately calculate the sum of $\exp\{-\frac{2\pi i}{S}(\sum_x^3 \delta_x w_x)\}$ over sets A_1, A_4, A_5, A_6, and the results are summarized in Table II.

TABLE II: The sum of $\exp\{-\frac{2\pi i}{S}(\sum_x^3 \delta_x w_x)\}$ over sets A_1, $A_2 \cup A_3$, A_4, A_5, A_6 for different strategy combinations of three individuals.

	$\delta_1 = \delta_2 = \delta_3$	$\delta_1 = \delta_2 \neq \delta_3$	$\delta_1 = \delta_3 \neq \delta_2$	$\delta_2 = \delta_3 \neq \delta_1$	$\delta_1 \neq \delta_2 \neq \delta_3 \neq \delta_1$
$\sum_{(w_1,w_2,w_3)\in A_1} \exp\{-\frac{2\pi i}{S}(\sum_x^3 \delta_x w_x)\}$	1	1	1	1	1
$\sum_{(w_1,w_2,w_3)\in(A_2\cup A_3)} \exp\{-\frac{2\pi i}{S}(\sum_x^3 \delta_x w_x)\}$	$(S - 1)(S - 2)$	$-S + 2$	$-S + 2$	$-S + 2$	2
$\sum_{(w_1,w_2,w_3)\in A_4} \exp\{-\frac{2\pi i}{S}(\sum_x^3 \delta_x w_x)\}$	$S - 1$	-1	-1	$S - 1$	-1
$\sum_{(w_1,w_2,w_3)\in A_5} \exp\{-\frac{2\pi i}{S}(\sum_x^3 \delta_x w_x)\}$	$S - 1$	-1	$S - 1$	-1	-1
$\sum_{(w_1,w_2,w_3)\in A_6} \exp\{-\frac{2\pi i}{S}(\sum_x^3 \delta_x w_x)\}$	$S - 1$	$S - 1$	-1	-1	-1

Substituting Tabel I and Tabel II into Eq. (11) and using $L_3(\cdot)+2L_1(\cdot)\Psi_1(\cdot)=3\Psi_1(\cdot)$, $L_4(\cdot)+2L_2(\cdot)\Psi_2(\cdot)=3\Psi_2(\cdot)$ (the sign $\cdot$ means $f(x)$), we have

$$Pr(s_1=\delta_1,s_2=\delta_2,s_3=\delta_3,h_2\cdot h_3=1)=\frac{1}{3MS^3}\sum_{x=1}^{M}$$

$$\begin{cases} 3\Psi_1(\cdot)+(S-1)(S-2)(2\Phi_1(\cdot)\Psi_2(\cdot)+\Phi_4(\cdot)\alpha_1)+(S-1)(3\Psi_2(\cdot)+2\Phi_2(\cdot)\Psi_2(\cdot)+2\Phi_3(\cdot)\Psi_1(\cdot) \\ +2\Phi_5(\cdot)\alpha_1), \qquad \text{if } \delta_1=\delta_2=\delta_3; \\ 3\Psi_1(\cdot)+(-S+2)(2\Phi_1(\cdot)\Psi_2(\cdot)+\Phi_4(\cdot)\alpha_1)-3\Psi_2(\cdot)+(S-2)(\Phi_2(\cdot)\Psi_2(\cdot)+\Phi_3(\cdot)\Psi_1(\cdot) \\ +\Phi_5(\cdot)\alpha_1), \qquad \text{if } \delta_1=\delta_2\neq\delta_3 \text{ or if } \delta_1=\delta_3\neq\delta_1; \\ 3\Psi_1(\cdot)+(-S+2)(2\Phi_1(\cdot)\Psi_2(\cdot)+\Phi_4(\cdot)\alpha_1)+3(S-1)\Psi_2(\cdot)-2(\Phi_2(\cdot)\Psi_2(\cdot)+\Phi_3(\cdot)\Psi_1(\cdot) \\ +\Phi_5(\cdot)\alpha_1), \qquad \text{if } \delta_2=\delta_3\neq\delta_1; \\ 3\Psi_1(\cdot)+2(2\Phi_1(\cdot)\Psi_2(\cdot)+\Phi_4(\cdot)\alpha_1)-3\Psi_2(\cdot)-2(\Phi_2(\cdot)\Psi_2(\cdot)+\Phi_3(\cdot)\Psi_1(\cdot)+\Phi_5(\cdot)\alpha_1), \\ \qquad \text{if } \delta_1\neq\delta_2\neq\delta_3\neq\delta_1; \end{cases} \tag{25}$$

where $\Psi_1(f)=\frac{1-v(1-f)}{1+(N-1)v(1-f)}$, $\Psi_2(f)=\frac{(1-u)(1-v(1-f))}{1+(N-1)u+(N-1)(1-u)v(1-f)}$, $\Phi_1(f)=\frac{(1-u)(2-v(1-f))}{2+(N-2)u+\frac{2(N-2)(1-u)v}{3}(1-f)}$, $\Phi_2(f)=\frac{2-u-v(1-f)}{2+\frac{2(N-2)u}{3}+\frac{(N-2)(2-u)v}{3}(1-f)}$, $\Phi_3(f)=\frac{(1-u)(2-v(1-f))}{2+\frac{2(N-2)u}{3}+\frac{(N-2)(2-u)v}{3}(1-f)}$, $\Phi_4(f)=\frac{(1-u)(1-v(1-f))}{1+\frac{(N-2)u}{2}+\frac{(N-2)(1-u)v}{3}(1-f)}$, $\Phi_5(f)=\frac{(2-u)(1-v(1-f))}{2+\frac{2(N-2)u}{3}+\frac{(N-2)(2-u)v}{3}(1-f)}$, $\alpha_1=\frac{1-u}{1+(N-1)u}$.

IV. THE CALCULATION OF $Pr(s_1=\delta_1,s_2=\delta_2,s_3=\delta_3,h_2\cdot h_3=1)$ FOR ANY MUTATION PATTERN WHEN THE WRIGHT-FISHER PROCESS IS USED.

Consider two individuals (say I_1,I_2) who are chosen randomly and without replacement from the population. During one update step, no coalescence happens to them with probability $\frac{N-1}{N}$, and they coalesce with probability $\frac{1}{N}$. Therefore, their first coalescence occurs at generation T by looking backwards from present generation 0 with probability

$$\frac{1}{N}(1-\frac{1}{N})^{T-1}. \tag{26}$$

Due to the independence of migration and mutation happening to the same individual and the independence of migration or the independence of mutation occurring at different generations, the probability of $D_1\bigcap D_2$, where D_x denotes that there are g_x migration events and h_x mutation events along the lineage of I_x during the former T update steps between generation 0 and generation T, is

$$M(g_1,g_2;h_1,h_2)=\prod_{x=1}^{2}\binom{T}{g_x}\binom{T}{h_x}v^{g_x}(1-v)^{T-g_x}u^{h_x}(1-u)^{T-h_x}. \tag{27}$$

There are no approximations in using the coalescence theory above, and our result holds for any population sizes, migration probabilities, and mutation probabilities.

In our model, the location space is characterized by a one-dimensional lattice satisfying the periodic boundary condition, $a + jM = a$, where j is an integer. Given that the ancestor of I_x at generation T is located in η_x and until then g_x migration events occur along the lineage of I_x, the migration process along the lineage of I_x becomes a discrete random walk on the one-dimensional lattice. One-step migration paths and the corresponding probabilities describe the displacement distribution generated by a single step of the random walker. Consequently, the probability that I_x is located in γ_x is

$$Pr_M(\eta_x \xrightarrow{g_x} \gamma_x) = M^{-1} \sum_{r=1}^{M} (\sum_{\triangle} p(\triangle) \exp(2\pi i \triangle \cdot r/M))^{g_x} \exp(-2\pi i r \cdot (\gamma_x - \eta_x)/M), \tag{28}$$

where $p(\triangle)$ is the probability that a single-step migration results in a displacement of location, $\triangle$, and i is the imaginary unit satisfying $i^2 = -1$. The function $f(r) = \sum_{\triangle} p(\triangle) \exp(2\pi i \triangle \cdot r/M)$, which corresponds to the structure function of the random walk on the one-dimensional lattice with period M [2], carries the full information of the migration pattern. Eq. (28) describes essentially the probability that a random walker moves from one to any point after t steps and holds for all migration patterns. There are no approximations in the above calculations, and thus our result is appropriate for any group numbers. Similarly, the strategy space is described as a one-dimensional lattice satisfying $b+jS = b$, where j is an integer. Given that the ancestor of I_x at generation T uses strategy θ_x and until then h_x mutation events occur along the lineage of I_x, the mutation process along the lineage of I_x can be traced by a discrete random walk on such a one-dimensional lattice. Eventually, the probability that I_x uses strategy δ_x is

$$Pr_S(\theta_x \xrightarrow{h_x} \delta_x) = S^{-1} \sum_{r=1}^{S} (\sum_{\triangle} h(\triangle) \exp(2\pi i \triangle \cdot r/S))^{h_x} \exp(-2\pi i r \cdot (\delta_x - \theta_x)/S), \tag{29}$$

where $h(\triangle)$ is the probability that a single-step mutation results in the change of strategy, $\triangle$. The function $g(r) = \sum_{\triangle} h(\triangle) \exp(2\pi i \triangle \cdot r/S)$ describes the full information of the mutation pattern.

When I_1 and I_2 coalesce for the first time, the probability that the common ancestor is located in η_1 and uses strategy θ_1 is denoted by

$$Pr(s_1 = \theta_1; m_1 = \eta_1) = \frac{1}{MS}, \tag{30}$$

where $\eta_1 \in \{1, \cdots, M\}$ and $\theta_1 \in \{1, \cdots, S\}$. T (the time that it takes I_1 and I_2 to reach their first coalescence) takes on one of the values $1, 2, \cdots$. Meanwhile, g_1, h_1, g_2, h_2 (g_i and h_i are the

number of migration events and of mutation events along the lineage of I_x among T update steps respectively) take on one value between 0 and T (including the boundaries). Given the above conditions, according to Eqs. (28), (29), the probability that I_1, I_2 are located in γ_1, γ_2 respectively and use strategies δ_1, δ_2 respectively is

$$Pr_M(\eta_1 \xrightarrow{g_1} \gamma_1) Pr_M(\eta_1 \xrightarrow{g_2} \gamma_2) Pr_S(\theta_1 \xrightarrow{h_1} \delta_1) Pr_S(\theta_1 \xrightarrow{h_2} \delta_2). \tag{31}$$

By considering all possible strategies and locations of the common ancestor in Eq. (30), all possible values of T in Eq. (26) and of g_1, g_2, h_1, h_2 in Eq. (27), and weighting the system in all steady states by the steady-state probabilities, we have the probability that I_1, I_2 are located in γ_1, γ_2 respectively and use strategies δ_1, δ_2 respectively as

$$\begin{aligned} & Pr(s_1 = \delta_1, s_2 = \delta_2; m_1 = \gamma_1, m_2 = \gamma_2) \\ & = \sum_{\theta_1, \eta_1} P(s_1 = \theta_1; m_1 = \eta_1) \sum_{T=1}^{+\infty} \frac{1}{N}(1 - \frac{1}{N})^{T-1} \sum_{g_1=0}^{T} \sum_{h_1=0}^{T} \sum_{g_2=0}^{T} \sum_{h_2=0}^{T} M(g_1, g_2; h_1, h_2) \\ & Pr_M(\eta_1 \xrightarrow{g_1} \gamma_1) Pr_M(\eta_1 \xrightarrow{g_2} \gamma_2) Pr_S(\theta_1 \xrightarrow{h_1} \delta_1) Pr_S(\theta_1 \xrightarrow{h_2} \delta_2) \\ & = \frac{1}{M^2 S^2} \sum_{x_1+x_2=M \,\text{or}\, 2M} \sum_{y_1+y_2=S \,\text{or}\, 2S} \Psi'(f(x_1), f(x_2), g(y_1), g(y_2)) \exp\{-\frac{2\pi i}{M}(x_1 \cdot \gamma_1 + x_2 \cdot \gamma_2)\} \\ & \exp\{-\frac{2\pi i}{S}(y_1 \cdot \delta_1 + y_2 \cdot \delta_2)\}, \end{aligned} \tag{32}$$

where

$$\Psi'(f(x_1), f(x_2), g(y_1), g(y_2)) = \frac{(1-u+ug(y_1))(1-u+ug(y_2))(1-v+vf(x_1))(1-v+vf(x_2))}{N-(N-1)(1-u+ug(y_1))(1-u+ug(y_2))(1-v+vf(x_1))(1-v+vf(x_2))}. \tag{33}$$

Consider three individuals (say I_1, I_2, I_3) who are chosen randomly and without replacement from the population. During one update step, no coalescence happens to them with probability $\frac{(N-1)(N-2)}{N^2}$, two of them coalesce with probability $\frac{3(N-1)}{N^2}$, and all of them coalesce simultaneously with probability $\frac{1}{N^2}$. The first coalescence of the three individuals happens to two of them at generation T by looking backwards from present generation 0 with probability

$$\frac{3(N-1)}{N^2}(1 - \frac{3(N-1)}{N^2} - \frac{1}{N^2})^{T-1}. \tag{34}$$

The first coalescence of the three individuals happens to all of them at generation T with probability

$$\frac{1}{N^2}(1 - \frac{3(N-1)}{N^2} - \frac{1}{N^2})^{T-1}. \tag{35}$$

During the former T update steps between generation 0 and generation T, the probability of $D_1 \bigcap D_2 \bigcap D_3$, where D_x denotes that there are g_x migration events and h_x mutation events along the lineage of I_x, is

$$M(g_1, g_2, g_3; h_1, h_2, h_3) = \prod_{x=1}^{3} \binom{T}{g_x}\binom{T}{h_x} v^{g_x}(1 - v)^{T-g_x} u^{h_x}(1 - u)^{T-h_x}. \tag{36}$$

When I_1, I_2, I_3 have two ancestors at the moment of the first coalescence, the probability that the two ancestors are located in η_1, η_2 respectively and use strategies θ_1, θ_2 respectively is denoted by

$$Pr(s_1 = \theta_1, s_2 = \theta_2; m_1 = \eta_1, m_2 = \eta_2). \tag{37}$$

There are three possible pairs for I_1, I_2, I_3 to first coalesce into a common ancestor. We assume that the first coalescence happens to I_x, I_y in the following. For simplicity, the common ancestor of I_x, I_y at generation T is assumed to be located in η_1 and use strategy θ_1, and the ancestor of the third individual at generation T is assumed to be located in η_2 and use strategy θ_2. Given the above conditions, according to Eqs. (28), (29), the probability that the three individuals are located in $\gamma_1, \gamma_2, \gamma_3$ respectively and use strategies $\delta_1, \delta_2, \delta_3$ respectively is

$$Pr_M(\eta_1 \xrightarrow{g_x} \gamma_x) Pr_M(\eta_1 \xrightarrow{g_y} \gamma_y) \cdots Pr_S(\theta_1 \xrightarrow{h_x} \delta_x) Pr_S(\theta_1 \xrightarrow{h_y} \delta_y) \cdots . \tag{38}$$

When I_1, I_2, I_3 have one common ancestor at the moment of the first coalescence, the probability that the common ancestor is located in η_1 and uses strategy θ_1 is

$$Pr(s_1 = \theta_1; m_1 = \eta_1). \tag{39}$$

Given such conditions, from Eqs. (28), (29), the probability that they are located in $\gamma_1, \gamma_2, \gamma_3$ respectively and use strategies $\delta_1, \delta_2, \delta_3$ respectively is

$$Pr_M(\eta_1 \xrightarrow{g_1} \gamma_1) Pr_M(\eta_1 \xrightarrow{g_2} \gamma_2) Pr_M(\eta_1 \xrightarrow{g_3} \gamma_3) Pr_S(\theta_1 \xrightarrow{h_1} \delta_1) Pr_S(\theta_1 \xrightarrow{h_2} \delta_2) Pr_S(\theta_1 \xrightarrow{h_3} \delta_3). \tag{40}$$

Considering all possible strategies and locations of the two ancestors or the single ancestor at the first coalescence (in Eqs. (37), (39)), all possible first coalescence pairs (in Eq. (38)), all possible values of T (in Eqs. (34), (35)), all possible values of $g_1, g_2, g_3, h_1, h_2, h_3$ (in Eq. (36)), and weighting the system in all steady states by the steady-state probabilities, we have the probability that three randomly chosen (without replacement) individuals are located in $\gamma_1, \gamma_2, \gamma_3$ respectively

and use strategies $\delta_1, \delta_2, \delta_3$ respectively is

$$
\begin{aligned}
& Pr(s_1 = \delta_1, s_2 = \delta_2, s_3 = \delta_3; m_1 = \gamma_1, m_2 = \gamma_2, m_3 = \gamma_3) \\
& = \sum_{\theta_1,\theta_2,\eta_1,\eta_2} P(s_1 = \theta_1, s_2 = \theta_2; m_1 = \eta_1, m_2 = \eta_2) \sum_{T=1}^{+\infty} \tfrac{N-1}{N^2}(1 - \tfrac{3(N-1)}{N^2} - \tfrac{1}{N^2})^{T-1} \sum_{g_1,h_1=0}^{T} \sum_{g_2,h_2=0}^{T} \\
& \sum_{g_3,h_3=0}^{T} M(g_1,g_2,g_3;h_1,h_2,h_3)(Pr_M(\eta_1 \xrightarrow{g_1} \gamma_1)Pr_M(\eta_1 \xrightarrow{g_2} \gamma_2)\, Pr_S(\theta_1 \xrightarrow{h_1} \delta_1)\, Pr_S(\theta_1 \xrightarrow{h_2} \delta_2) \\
& Pr_M(\eta_2 \xrightarrow{g_3} \gamma_3)Pr_S(\theta_2 \xrightarrow{h_3} \delta_3) + Pr_M(\eta_1 \xrightarrow{g_1} \gamma_1)Pr_M(\eta_1 \xrightarrow{g_3} \gamma_3)Pr_S(\theta_1 \xrightarrow{h_1} \delta_1)Pr_S(\theta_1 \xrightarrow{h_3} \delta_3) \\
& Pr_M(\eta_2 \xrightarrow{g_2} \gamma_2)Pr_S(\theta_2 \xrightarrow{h_2} \delta_2) + Pr_M(\eta_1 \xrightarrow{g_2} \gamma_2)Pr_M(\eta_1 \xrightarrow{g_3} \gamma_3)Pr_S(\theta_1 \xrightarrow{h_2} \delta_2)Pr_S(\theta_1 \xrightarrow{h_3} \delta_3) \\
& Pr_M(\eta_2 \xrightarrow{g_1} \gamma_1)Pr_S(\theta_2 \xrightarrow{h_1} \delta_1)) + \sum_{\theta_1,\eta_1} P(s_1 = \theta_1; m_1 = \eta_1) \sum_{T=1}^{+\infty} \tfrac{1}{N^2}(1 - \tfrac{3(N-1)}{N^2} - \tfrac{1}{N^2})^{T-1} \sum_{g_1,h_1=0}^{T} \\
& \sum_{g_2,h_2=0}^{T} \sum_{g_3,h_3=0}^{T} M(g_1,g_2,g_3;h_1,h_2,h_3)Pr_M(\eta_1 \xrightarrow{g_1} \gamma_1)Pr_M(\eta_1 \xrightarrow{g_2} \gamma_2)\, Pr_S(\theta_1 \xrightarrow{h_1} \delta_1) \\
& Pr_S(\theta_1 \xrightarrow{h_2} \delta_2)Pr_M(\eta_1 \xrightarrow{g_3} \gamma_3)Pr_S(\theta_1 \xrightarrow{h_3} \delta_3) \\
& = \tfrac{1}{M^3S^3} \sum_{z_1+z_2+z_3=M,2M or 3M} \sum_{w_1+w_2+w_3=S,2S or 3S} \quad \{\sum_{x_1=z_1+z_2,x_2=z_3,y_1=w_1+w_2,y_2=w_3} + \sum_{x_1=z_1+z_3,x_2=z_2,} \\
& {}_{y_1=w_1+w_3,y_2=w_2} + \sum_{x_1=z_2+z_3,x_2=z_1,y_1=w_2+w_3,y_2=w_1}\}(N-1)\Phi'(f(z_1), f(z_2), f(z_3), g(w_1), g(w_2), g(w_3))\times \\
& \Psi'(f(x_1), f(x_2), g(y_1), g(y_2)) \exp\{-\tfrac{2\pi i}{M}(z_1 \cdot \gamma_1 + z_2 \cdot \gamma_2 + z_3 \cdot \gamma_3)\} \exp\{-\tfrac{2\pi i}{S}(w_1 \cdot \delta_1 + w_2 \cdot \delta_2 \\
& + w_3 \cdot \delta_3)\} + \tfrac{1}{M^3S^3} \sum_{z_1+z_2+z_3=M,2M or 3M} \sum_{w_1+w_2+w_3=S,2S or 3S} \Phi'(f(z_1), f(z_2), f(z_3), g(w_1), g(w_2), g(w_3)) \\
& \exp\{-\tfrac{2\pi i}{M}(z_1 \cdot \gamma_1 + z_2 \cdot \gamma_2 + z_3 \cdot \gamma_3)\} \exp\{-\tfrac{2\pi i}{S}(w_1 \cdot \delta_1 + w_2 \cdot \delta_2 + w_3 \cdot \delta_3)\},
\end{aligned}
\tag{41}
$$

where

$$
\Phi'(f(z_1), f(z_2), f(z_3), g(w_1), g(w_2), g(w_3)) = \frac{\prod_{i=1}^{3}(1-u+ug(w_i))(1-v+vf(z_i))}{N^2-(N-2)(N-1)\prod_{i=1}^{3}(1-u+ug(w_i))(1-v+vf(z_i))}. \tag{42}
$$

The probability that three randomly chosen (without replacement) individuals (say I_1, I_2, I_3 or $1, 2, 3$) satisfy $s_1 = \delta_1, s_2 = \delta_2, s_3 = \delta_3, h_2 \cdot h_3 = 1$ is

$$
\begin{aligned}
& Pr(s_1 = \delta_1, s_2 = \delta_2, s_3 = \delta_3, h_2 \cdot h_3 = 1) \\
& = \sum_{\gamma_1=1}^{M} \sum_{\gamma_2=\gamma_3=1}^{M} P(s_1 = \delta_1, s_2 = \delta_2, s_3 = \delta_3; m_1 = \gamma_1, m_2 = \gamma_2, m_3 = \gamma_3) \\
& = \tfrac{1}{MS^3} \sum_{z_1=M,z_2+z_3=M or 2M} \sum_{w_1+w_2+w_3=S,2S or 3S} \quad \{\sum_{x_1=z_1+z_2,x_2=z_3,y_1=w_1+w_2,y_2=w_3} + \sum_{x_1=z_1+z_3,x_2=z_2,} \\
& {}_{y_1=w_1+w_3,y_2=w_2} + \sum_{x_1=z_2+z_3,x_2=z_1,y_1=w_2+w_3,y_2=w_1}\}((N-1)\Phi'(f(z_1), f(z_2), f(z_3), g(w_1), g(w_2), g(w_3))\times \\
& \Psi'(f(x_1), f(x_2), g(y_1), g(y_2)) \exp\{-\tfrac{2\pi i}{S}(w_1 \cdot \delta_1 + w_2 \cdot \delta_2 + w_3 \cdot \delta_3)\} + \tfrac{1}{MS^3} \sum_{z_1=M,z_2+z_3=M or 2M} \sum_{w_1} \\
& {}_{+w_2+w_3=S,2S or 3S} \Phi'(f(z_1), f(z_2), f(z_3), g(w_1), g(w_2), g(w_3)) \exp\{-\tfrac{2\pi i}{S}(w_1 \cdot \delta_1 + w_2 \cdot \delta_2 + w_3 \cdot \delta_3)\}.
\end{aligned}
\tag{43}
$$

The equal sign holds because $\sum_{\gamma_1=1}^{M}$ and $\sum_{\gamma_2=\gamma_3=1}^{M}$ mean $(N-1)\Phi'(\cdots) \times \Psi'(\cdots)$ or $\Phi'(\cdots)$ does not vanish only if $z_1 = M, z_2 + z_3 = M, \text{or}\, 2M$.

V. THE CALCULATION OF $Pr(s_1 = \delta_1, s_2 = \delta_2, s_3 = \delta_3, h_2 \cdot h_3 = 1)$ FOR 'GLOBAL MUTATION' WHEN THE WRIGHT-FISHER PROCESS IS USED.

Substituting Eqs. (14), (15) into Eq.(42), we have

$$\Phi'(\cdots) = \begin{cases} \Phi'_1(f(z_2)) = \frac{(1-v+vf(z_2))^2}{N^2-(N-2)(N-1)(1-v+vf(z_2))^2}, & \text{if } (w_1, w_2, w_3) \in A_1; \\ \Phi'_2(f(z_2)) = \frac{(1-u)^3(1-v+vf(z_2))^2}{N^2-(N-2)(N-1)(1-u)^3(1-v+vf(z_2))^2}, & \text{if } (w_1, w_2, w_3) \in A_2 \cup A_3; \\ \Phi'_3(f(z_2)) = \frac{(1-u)^2(1-v+vf(z_2))^2}{N^2-(N-2)(N-1)(1-u)^2(1-v+vf(z_2))^2}, & \text{if } (w_1, w_2, w_3) \in A_4 \cup A_5 \cup A_6. \end{cases} \tag{44}$$

For $x_1 = z_1 + z_2, x_2 = z_3, y_1 = w_1 + w_2, y_2 = w_3$, substituting Eq. (16) into Eq. (33), we have

$$\Psi'(\cdots) = \begin{cases} L'_1(f(z_2)) = \frac{(1-v+vf(z_2))^2}{N-(N-1)(1-v+vf(z_2))^2}, & \text{if } (w_1, w_2, w_3) \in A_1 \cup A_6; \\ L'_2(f(z_2)) = \frac{(1-u)^2(1-v+vf(z_2))^2}{N-(N-1)(1-u)^2(1-v+vf(z_2))^2}, & \text{if } (w_1, w_2, w_3) \in A_2 \cup A_3 \cup A_4 \cup A_5. \end{cases} \tag{45}$$

For $x_1 = z_1 + z_3, x_2 = z_2, y_1 = w_1 + w_3, y_2 = w_2$, substituting Eq. (19) into Eq. (33), we have

$$\Psi'(\cdots) = \begin{cases} L'_1(f(z_2)), & \text{if } (w_1, w_2, w_3) \in A_1 \cup A_5; \\ L'_2(f(z_2)), & \text{if } (w_1, w_2, w_3) \in A_2 \cup A_3 \cup A_4 \cup A_6. \end{cases} \tag{46}$$

For $x_1 = z_2 + z_3, x_2 = z_1, y_1 = w_2 + w_3, y_2 = w_1$, substituting Eq. (22) into Eq. (33), we have

$$\Psi'(\cdots) = \begin{cases} 1, & \text{if } (w_1, w_2, w_3) \in A_1 \cup A_4; \\ L'_3 = \frac{(1-u)^2}{N-(N-1)(1-u)^2}, & \text{if } (w_1, w_2, w_3) \in A_2 \cup A_3 \cup A_5 \cup A_6. \end{cases} \tag{47}$$

According to Eqs. (44), (45), (46), (47), the expressions of $(N-1)\Phi'(\cdots)\,\Psi'(\cdots)$ for the points $(w_1, w_2, w_3) \in (\cup_{x=1}^{6} A_x)$ and different coalescence combinations are summarized in Table III.

Let $(N-1)L'_1(\cdot) + 1 = \frac{N}{N-(N-1)(1-v+vf(x))^2} = N\Psi'_1(\cdot)$, $(N-1)L'_2(\cdot) + 1 = \frac{N}{N-(N-1)(1-u)^2(1-v+vf(x))^2} = N\Psi'_2(\cdot)$, $(N-1)L'_3 + 1 = \frac{N}{N-(N-1)(1-u)^2} = \alpha'_1$, and then we have

$2(N-1)\Phi'_1(\cdot)L'_1(\cdot) + N\Phi'_1(\cdot) = \Phi'_1(\cdot)(2N\Psi'_1(\cdot) + N - 2)$,

$2(N-1)\Phi'_2(\cdot)L'_2(\cdot) + (N-1)\Phi'_2(\cdot)L'_3 + \Phi'_2(\cdot) = \Phi'_2(\cdot)(2N\Psi'_2(\cdot) + N\alpha'_1 - 2)$,

$2(N-1)\Phi'_3(\cdot)L'_2(\cdot) + N\Phi'_3(\cdot) = \Phi'_3(\cdot)(2N\Psi'_2(\cdot) + N - 2)$,

$(N-1)\Phi'_3(\cdot)L'_2(\cdot) + (N-1)\Phi'_3(\cdot)L'_1(\cdot) + (N-1)\Phi'_3(\cdot)L'_3 + \Phi'_3(\cdot) = \Phi'_3(\cdot)(N\Psi'_2(\cdot) + N\Psi'_1(\cdot) + N\alpha'_1 - 2)$.

TABLE III: The expressions of $(N-1)\Phi'(\cdots)\Psi'(\cdots)$ for the points $(w_1,w_2,w_3)\in(\cup_{x=1}^{6}A_x)$ and different coalescence combinations.

	$x_1=z_1+z_2, x_2=z_3$ $y_1=w_1+w_2, y_2=w_3$	$x_1=z_1+z_3, x_2=z_2$ $y_1=w_1+w_3, y_2=w_2$	$x_1=z_2+z_3, x_2=z_1$ $y_1=w_2+w_3, y_2=w_1$
$(w_1,w_2,w_3)\in A_1$	$(N-1)\Phi_1'(\cdot)L_1'(\cdot)$	$(N-1)\Phi_1'(\cdot)L_1'(\cdot)$	$(N-1)\Phi_1'(\cdot)$
$(w_1,w_2,w_3)\in(A_2\cup A_3)$	$(N-1)\Phi_2'(\cdot)L_2'(\cdot)$	$(N-1)\Phi_2'(\cdot)L_2'(\cdot)$	$(N-1)\Phi_2'(\cdot)L_3'$
$(w_1,w_2,w_3)\in A_4$	$(N-1)\Phi_3'(\cdot)L_2'(\cdot)$	$(N-1)\Phi_3'(\cdot)L_2'(\cdot)$	$(N-1)\Phi_3'(\cdot)$
$(w_1,w_2,w_3)\in A_5$	$(N-1)\Phi_3'(\cdot)L_2'(\cdot)$	$(N-1)\Phi_3'(\cdot)L_1'(\cdot)$	$(N-1)\Phi_3'(\cdot)L_3'$
$(w_1,w_2,w_3)\in A_6$	$(N-1)\Phi_3'(\cdot)L_1'(\cdot)$	$(N-1)\Phi_3'(\cdot)L_2'(\cdot)$	$(N-1)\Phi_3'(\cdot)L_3'$

Combining Table III and Table II and using the above equalities, we have

$$Pr(s_1=\delta_1,s_2=\delta_2,s_3=\delta_3,h_2\cdot h_3=1)=\frac{1}{S^3M}\sum_{x=1}^{M}$$

$$\begin{cases}\Phi_1'(\cdot)(2N\Psi_1'(\cdot)+N-2)+(S-1)(S-2)\Phi_2'(\cdot)(2N\Psi_2'(\cdot)+N\alpha_1'-2)+(S-1)\Phi_3'(\cdot)(2N\Psi_2'(\cdot)\\ +N-2)+2(S-1)\Phi_3'(\cdot)(N\Psi_2'(\cdot)+N\Psi_1'(\cdot)+N\alpha_1'-2) \qquad \text{if } \delta_1=\delta_2=\delta_3,\\ \Phi_1'(\cdot)(2N\Psi_1'(\cdot)+N-2)+(-S+2)\Phi_2'(\cdot)(2N\Psi_2'(\cdot)+N\alpha_1'-2)-\Phi_3'(\cdot)(2N\Psi_2'(\cdot)+N-2)\\ +(S-2)\Phi_3'(\cdot)(N\Psi_2'(\cdot)+N\Psi_1'(\cdot)+N\alpha_1'-2), \qquad \text{if } \delta_1=\delta_2\neq\delta_3,\ \delta_1=\delta_3\neq\delta_2,\\ \Phi_1'(\cdot)(2N\Psi_1'(\cdot)+N-2)+(-S+2)\Phi_2'(\cdot)(2N\Psi_2'(\cdot)+N\alpha_1'-2)+(S-1)\Phi_3'(\cdot)(2N\Psi_2'(\cdot)\\ +N-2)-2\Phi_3'(\cdot)(N\Psi_2'(\cdot)+N\Psi_1'(\cdot)+N\alpha_1'-2), \qquad \text{if } \delta_2=\delta_3\neq\delta_1,\\ \Phi_1'(\cdot)(2N\Psi_1'(\cdot)+N-2)+2\Phi_2'(\cdot)(2N\Psi_2'(\cdot)+N\alpha_1'-2)-\Phi_3'(\cdot)(2N\Psi_2'(\cdot)+N-2)-2\Phi_3'(\cdot)\\ (N\Psi_2'(\cdot)+N\Psi_1'(\cdot)+N\alpha_1'-2), \qquad \text{if } \delta_1\neq\delta_2\neq\delta_3\neq\delta_1,\end{cases} \tag{48}$$

where $\alpha_1'=\frac{1}{N-(N-1)(1-u)^2}$, $\Phi_1'(f)=\frac{(1-v(1-f))^2}{N^2-(N-1)(N-2)(1-v(1-f))^2}$, $\Phi_2'(f)=\frac{(1-u)^3(1-v(1-f))^2}{N^2-(N-1)(N-2)(1-u)^3(1-v(1-f))^2}$, $\Phi_3'(f)=\frac{(1-u)^2(1-v(1-f))^2}{N^2-(N-1)(N-2)(1-u)^2(1-v(1-f))^2}$, $\Psi_1'(f)=\frac{1}{N-(N-1)(1-v(1-f))^2}$, $\Psi_2'(f)=\frac{1}{N-(N-1)(1-u)^2(1-v(1-f))^2}$.

VI. MIGRATION OCCURS BEFORE REPRODUCTION.

In the main text, we focus on the model in which migration occurs after reproduction. Here, we consider a second case in which migration occurs before reproduction and the rest follows

from the model in the main text. Specifically, one individual is chosen equi-probably (from the whole population) to migrate before reproduction in the Moran process, and all individuals migrate before reproduction in the Wright-Fisher process. By Monte Carlo simulations, we compare the results of the two cases based on the values of σ_1 and σ_2 both for the Moran process (Fig. 1) and for the Wright-Fisher process (Fig. 2). The two cases have a small or even a negligible difference irrespective of the mutation probability when the migration probability is small, and the difference becomes larger as the migration probability increases. However, σ_1 or σ_2 has the same monotonicity with respect to the mutation probability and the migration probability in the two cases. Accordingly, the results of the two cases are qualitatively similar but quantitatively different. The quantitative difference emerges because mutation and migration in the second case can not happen to the same individual during a generation, which happens in the initial case.

---

[1] Zhang, Y. L., Fu, F., Chen, X. J., Xie, G. M. & Wang, L. Cooperation in group-structured populations with two layers of interactions. *Scientific Reports* **5,** 17446 (2015).

[2] Montroll, E. W. & Weiss, G. H. Random Walks on Lattices. II *J. Math. Phys.* **6,** 167–181 (1965).

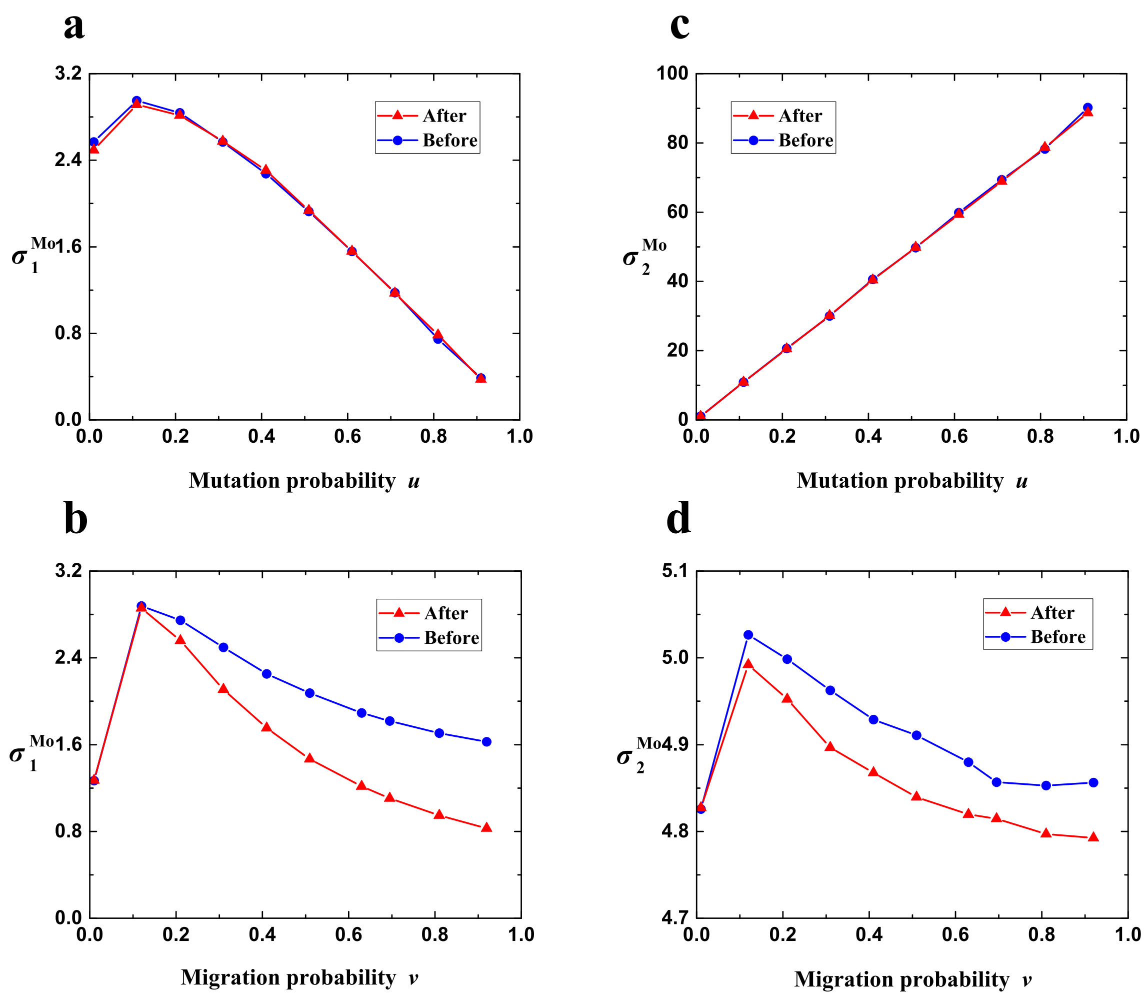

FIG. 1: Comparison of two cases in which migration occurs after or before reproduction when the Moran process is used. For low $v = 0.1$, σ_1 (**a**) or σ_2 (**c**) of the 'after' case (in which migration occurs after reproduction) is almost identical to that of the 'before' case (in which migration occurs before reproduction) irrespective of u. In the two cases, σ_1 or σ_2 has the same monotonicity with respect to u. As v increases, σ_1 (**b**) or σ_2 (**d**) of the 'after' case has a larger difference from that of the 'before' case. In the two cases, σ_1 or σ_2 has the same monotonicity with respect to v. Parameters: (**a**, **c**) $v = 0.1$, $N = 100$, $M = 19$, $r = 1$; (**b**, **d**) $u = 0.1$, $N = 50$, $M = 9$, $r = 4$.

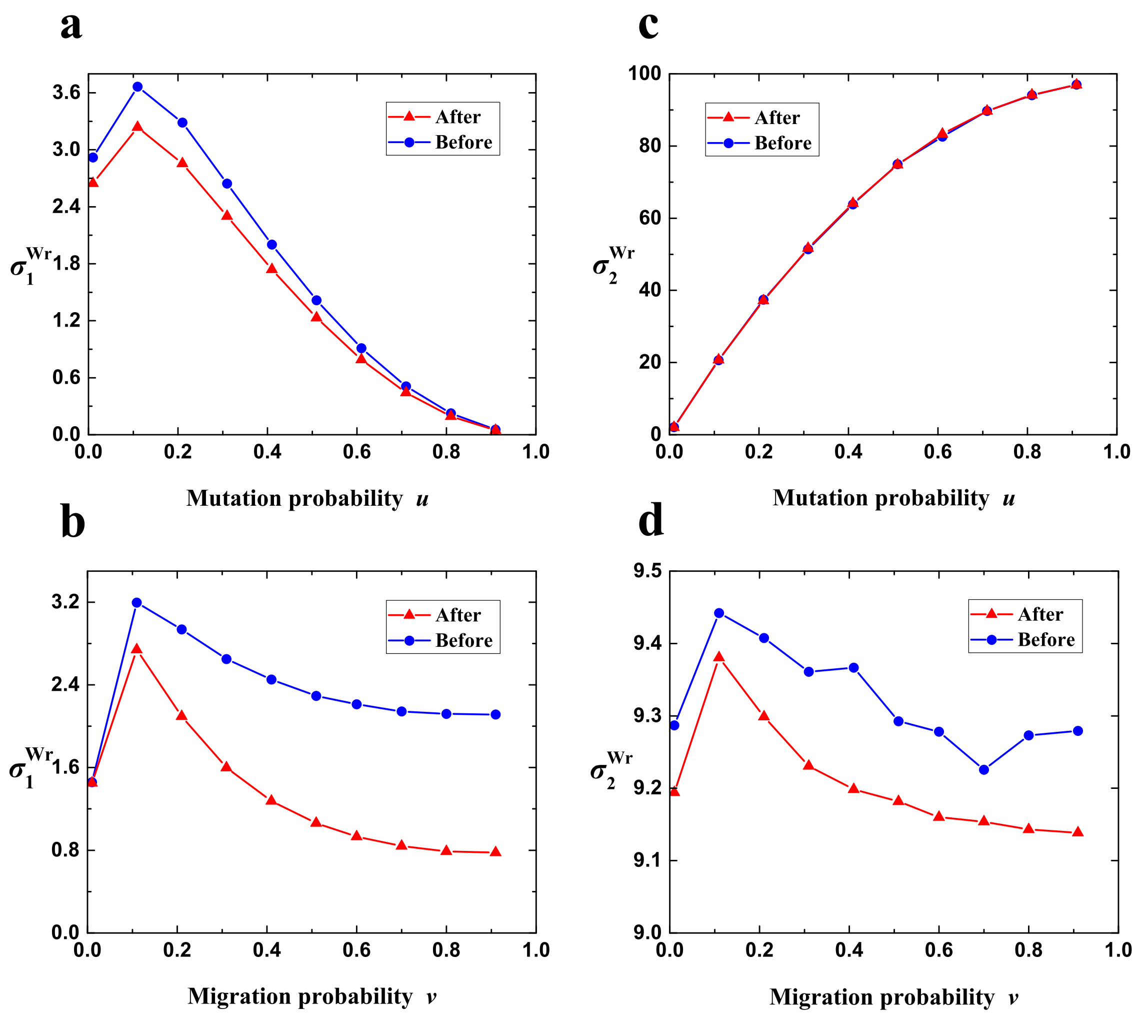

FIG. 2: Comparison of two cases in which migration occurs after or before reproduction when the Wright-Fisher process is used. For low $v = 0.1$, σ_1 (**a**) or σ_2 (**c**) of the 'after' case (in which migration occurs after reproduction) is slightly different from or almost identical to that of the 'before' case (in which migration occurs before reproduction) irrespective of u. In the two cases, σ_1 or σ_2 has the same monotonicity with respect to u. As v increases, σ_1 (**b**) or σ_2 (**d**) of the 'after' case has a larger difference from that of the 'before' case. In the two cases, σ_1 or σ_2 has the same monotonicity with respect to v. Parameters: (**a**, **c**) $v = 0.1$, $N = 100$, $M = 19$, $r = 1$; (**b**, **d**) $u = 0.1$, $N = 50$, $M = 9$, $r = 4$.
